# A systematic review and meta-analysis on dual-task sensor-based motion analysis for dementia detection

**DOI:** 10.3389/fdgth.2026.1728588

**Published:** 2026-05-07

**Authors:** Iman Hosseini, Joseph M. Northey, Nathan M. D’Cunha, Raul Fernandez Rojas, Abishek Shrestha, Maryam Ghahramani

**Affiliations:** 1 School of Computing, Australian National University, Acton, ACT, Australia; 2 UC Research Institute for Sport and Exercise, University of Canberra, Bruce, ACT, Australia; 3 Centre for Ageing Research & Translation, University of Canberra, Bruce, ACT, Australia; 4 BioSIS (Biosensing & Intelligent Systems) Lab, Centre for Intelligent Computing and Systems, University of Canberra, Canberra, ACT, Australia

**Keywords:** cognitive-motor interference, dementia detection, digital biomarkers, dual-task assessment, gait analysis, instrumented analysis

## Abstract

**Introduction:**

Early diagnosis of dementia may be improved by objective, scalable tests that capture how cognitive tasks interfere with movement. This study examined the use of instrumented dual-task paradigms for dementia detection and characterisation.

**Methods:**

We performed a PRISMA-guided systematic review and meta-analysis of peer-reviewed studies that used dual-task paradigms in adults with clinically defined dementia and an appropriate comparator. We extracted primary motor tasks, secondary cognitive or motor loads, sensor modalities, and analytic approaches. Walking outcomes were meta-analysed using inverse-variance weighted random-effects models, including subgroup analyses for single-task versus dual-task conditions and for arithmetic versus memory and verbal fluency assessments.

**Results:**

The literature was dominated by cognitive-motor dual-task paradigms in Alzheimer's disease cohorts. Inertial measurement units and force plates were the most common instruments, and most studies used classical statistics, with fewer applying machine learning. Pooled effects showed consistent group differences; compared with controls, people with dementia walked more slowly, took shorter steps, and showed less steady timing. Although heterogeneity was substantial across studies, the direction of effects was stable, and dual-task conditions generally amplified group differences relative to single-task performance. Arithmetic loads tended to accentuate changes linked to speed and cadence, whereas memory and verbal fluency assessments tended to prolong timing measures. Balance, turning, and some upper-limb outcomes also differentiated groups.

**Discussion:**

Instrumented dual-task assessments appear to enhance detection of cognitive-motor impairment in dementia and may complement existing evaluations. To support clinical translation, future work should extend beyond Alzheimer's disease, standardise task instructions and reporting, and evaluate multi-modal, validated analytic approaches across different dementia subtypes.

**Systematic Review Registration:**

https://www.crd.york.ac.uk/PROSPERO/view/CRD420251114199, CRD420251114199.

## 1 Introduction

Dementia is a progressive neurodegenerative syndrome that leads to a gradual decline in cognitive abilities and impairment in daily functioning. As the condition advances, people with dementia not only experience memory loss and deficits in other cognitive domains but also a gradual decline of independence. An essential component of quality of life in older adults is functional autonomy, and the loss of this independence due to dementia is associated with increased rates of hospitalisation, entry into residential aged care, and mortality [1]. In parallel with cognitive deterioration, physical impairments such as gait symptoms, balance deficits, and postural instability often emerge, contributing to a heightened risk of falls and further loss of functional capacity [2]. The severity and onset of functional impairments vary across individuals, influenced by factors such as dementia subtype, sex, and educational background [3]. Understanding the interplay between cognitive and functional deterioration is crucial for developing interventions aimed at preserving independence and improving quality of life for people living with dementia.

People with dementia experience significant alterations in brain function that affect cognitive and consequently motor performance. The neurodegenerative process leads to structural and functional changes in key brain regions such as the hippocampus, basal ganglia, and prefrontal cortex [4]. Irrespective of subtype, dementia affects movement control in the brain, which results in changed walking patterns, reduced speed, and impaired balance.

The deterioration of brain regions involved in motor control leads to disruptions in mobility, affecting an individual’s ability to walk safely and maintain postural stability. People with dementia often experience altered gait patterns [5] and reduced walking speed [6, 7]. These motor dysfunctions are not merely physical limitations but rather a consequence of the cognitive decline that disrupts the complex neural networks responsible for coordinating movement [8]. Walking is a cognitively demanding task that requires continuous sensory integration, spatial awareness, motor planning, and real-time adjustments, all of which are impaired in dementia. As executive functions deteriorate, people with dementia struggle with divided attention and adaptive control, further compounding mobility impairments [9]. Balance deficits are also pronounced, as dementia-related neurodegeneration affects proprioception, vestibular function, and postural reflexes, increasing fall risk and reducing stability during movement. Additionally, changes in specific gait parameters, such as increased stride time and higher stride variability, have been observed in people with dementia and cognitive impairment, suggesting their potential as early biomarkers for disease progression [10]. Given the significant cognitive load required for motor function, the decline in both cognitive and neural integrity in dementia severely impacts mobility, balance, and overall movement coordination.

Dual-tasking paradigms have emerged as a valuable approach to assess interactions between cognitive and motor functions in dementia and may complement existing diagnostic methods [11]. These tasks usually involve performing a motor activity, such as walking, while simultaneously engaging in a secondary cognitive task (e.g., mathematical operations or verbal fluency tests). Dual-tasking performance declines significantly in individuals with cognitive impairment, with more pronounced differences observed compared to single-task performance [12–14]. The effort required to maintain performance in dual-task scenarios, known as dual-task costs, is significantly higher in individuals with dementia, reflecting the cognitive strain and the competition for shared neural resources [1, 15]. Given the direct impact of cognitive load on gait parameters and stability, dual-task assessments hold promise as a predictive tool for the early detection of mild cognitive impairment (MCI) and dementia.

Different types of dual-tasking, when combined with sensor-based assessments, provide valuable insights into how dementia affects cognitive and motor performance. Dual motor tasks involve performing two motor activities simultaneously, such as walking while carrying an object, which challenges coordination and balance [16]. Cognitive–motor dual-task paradigms, the most relevant for dementia research, involve a combination of a motor task and a cognitive task, such as walking while counting backward. These tasks place significant demands on cognitive resources, requiring the brain to allocate attention effectively between motor and cognitive processes [17]. By integrating sensor-based measurements, researchers can quantify cognitive-motor interference with greater precision. This will help detect early markers of dementia that may not be evident through traditional clinical assessments. Given such ability to provide non-invasive, cost-effective, and objective evaluations, dual-task assessments combined with sensor technology represent a promising approach for early diagnosis and intervention in dementia. This will stress the need to understand the role of cognitive and motor assessments in dementia detection, identifying key methodologies, critical task features, and analytical techniques used in research.

This systematic review builds upon existing research on the role of dual-task assessments in dementia detection. Our primary objectives are to answer the following research questions (RQs):

1. **RQ1: What types of dual-task assessments are being used in dementia research?**
  This question aims to identify and categorise the various dual-task paradigms used in dementia detection studies, including dual-motor tasks and cognitive-motor dual-task paradigms. By analysing the tasks used in each category, our review also seeks to understand how task prioritisation is managed by individuals with dementia and how different dual-task combinations may affect performance outcomes.
2. **RQ2: What types and methods of assessment are being used, and what features are extracted from each physical and cognitive task?**
  This question focuses on the tools and methodologies used to evaluate gait, balance, motion, and cognition during dual-task performance. It includes an examination of the paradigms and mechanisms used for assessment, the role of various measurement devices (e.g., inertial measurement units (IMUs), force plates) in capturing objective data, and the specific quantitative features extracted from both primary and secondary tasks. This will help understand dual-tasks structures and the types of data used in dementia detection.
3. **RQ3: What analytical methods and mechanisms are used to interpret the results of dual- task assessments?**
  This question aims to investigate the analytical approaches applied in the interpretation of dual-task performance data. It includes statistical methods, traditional machine learning algorithms, and deep learning techniques. This will help explore how these methods are currently being used to detect dementia-related conditions and evaluate their effectiveness and possible limitations.
4. **RQ4: Are there specific tasks and/or features within the dual-task assessments that are particularly effective in detecting dementia?**
  This question aims to identify which dual-task paradigms and their corresponding extracted quantitative features are the most predictive of dementia, and whether features derived from dual-task performance offer greater discriminatory power for dementia detection than those extracted from single-task assessments.

We will examine studies that assess dual-task performance in people with different types of dementia, with a particular focus on its effects on motor function and cognitive performance. Additionally, we explore the potential of advanced sensor-based motion assessments to enhance detection strategies. This review highlights the dual-task paradigms, assessment mechanisms, and extracted features that most consistently distinguish people with dementia from healthy people. By synthesising these findings, the results will help guide future research and clinical practice toward developing more targeted, data-driven, and reliable dementia diagnostic protocols.

## 2 Related works

Among the currently published literature, there are four relevant systematic reviews that explore dual-task assessments in cognitive impairment and dementia. Table 1 summarises these studies, highlighting their main focus, the number of papers reviewed, time span, and their limitations relative to the aims of the our review. For example, Longhurst et al. [18] provided a broad overview of cognitive-motor interference across neurodegenerative conditions, including Alzheimer’s and Parkinson’s disease, but did not systematically classify dual-task paradigms or examine analytical tools and feature effectiveness in dementia detection. While each review contributes useful insights into motion and cognitive assessment strategies, none provide a comprehensive and integrated analysis covering dual-task typologies, assessment methods, extracted features, analytical mechanisms, and predictive utility for dementia, which are key aspects that the our review aims to address.

**Table 1:** Comparison of existing systematic reviews with respect to our research aims.

Study	Main focus	# papers reviewed	Year range	Limitations
Bishnoi and Hernandez [19]	Systematic review and meta-analysis of dual-task walking costs in MCI vs. healthy controls, focusing on walking speed under various cognitive tasks.	20	Up to 2020	∙ Focused only on gait speed changes under dual-task conditions.
				∙ No systematic discussion of task categorisation or prioritisation (RQ1).
				∙ Sensors, extracted features, or cognitive task metrics not covered (RQ2).
				∙ No analysis of ML/statistical methods used to interpret results (RQ3).
				∙ Identifies effective dual-task paradigms (e.g., serial subtraction and verbal fluency), but not feature-level predictive value (RQ4).
Longhurst et al. [18]	Broad scoping review of cognitive-motor interference in AD, PD, MCI using dual-task paradigms	95	1990s–2020	∙ Categorises cognitive and motor tasks but not clearly classified into paradigms
				∙ Extracted features and sensors not systematically reviewed
				∙ Analytical methods briefly discussed but not systematically compared
				∙ Does not identify which tasks/features are most predictive
Mancioppi et al. [20]	Focuses on cognitive dual-task protocols and technologies used in MCI populations	38	2010–2020	∙ Strong on task types and protocols, but no prioritisation strategies discussed
				∙ Lacks analysis of data interpretation methods
				∙ Does not evaluate which tasks/features are most predictive
Aditya et al. [21]	Focuses on gait parameters in dementia/MCI during single vs dual-task protocols	23	2014–2024	∙ Task types and gait measures discussed but not fully categorised
				∙ Only focuses on gait parameters, sensors/technologies not systematically compared
				∙ Analytical methods not reviewed

## 3 Methods

### 3.1 Review protocol and registration

This systematic review and meta-analysis followed the guidelines provided by the Preferred Reporting Items for Systematic Reviews and Meta-Analyses (PRISMA) statement [22] and was registered in the PROSPERO database (Registration ID: CRD420251114199). The methodology was pre-determined and documented in a protocol that was rigorously followed to ensure consistency and transparency in the review process. The review was conducted in accordance with the registered PROSPERO, with no substantial deviations.

To ensure a structured and comprehensive approach, the PICOS (Population, Intervention, Comparator, Outcomes, and Study Design) framework [23] was used to formulate the research question and, subsequently, to define the inclusion and exclusion criteria for this systematic review.

### 3.2 Search strategy

A comprehensive search was conducted across four electronic databases: Scopus, PubMed, IEEE Xplore, and Web of Science. The search terms used included: (“dementia” OR “Alzheimer’s disease” OR “YOD” OR “Creutzfeldt–Jakob disease” OR “Vascular” OR “Frontotemporal” OR “Lewy body” OR “DLB” OR “cognitive-motor interference”) AND “dual-task*” AND (“machine learning” OR “deep learning” OR “classify*” OR “diagnose*” OR “detect*”). The search was limited to studies published between 2014 and 2025. The initial literature search was conducted in late 2024. Following manuscript submission and peer-review in 2025, the search was updated prior to resubmission to ensure inclusion of the most recent evidence. Consequently, the final review includes studies published between 2014 and 2025. Additionally, reference lists of all included articles were screened and any potentially relevant studies identified through citation searching were assessed for eligibility and included if they met this review’s inclusion criteria.

### 3.3 Eligibility criteria

This review included peer-reviewed journal articles published between 2014 and 2025 and written in English. The included studies employed dual-motor and dual cognitive-motor assessments, where participants performed either secondary motor task or a cognitive task simultaneously with other motion and gait-related activities. Studies that examined only functional assessments without a dual-task motor assessment component were excluded.

The included studies are required to perform comparative analyses. Acceptable comparators involved either: (1) comparisons between individuals with clinically diagnosed dementia of any subtypes (Alzheimer’s disease (AD), vascular dementia (VD), frontotemporal dementia, dementia with Lewy bodies (DLB), Creutzfeldt–Jakob disease (CJD), and general dementia) and other participant groups, such as healthy controls with normal cognition, individuals with mild cognitive impairment (MCI), or, where appropriate, individuals with Parkinson’s disease (PD); and/or (2) comparisons between different assessment conditions, such as dual-task vs. single-task performance. Studies that did not include a relevant comparator group to assess cognitive status or task performance differences were excluded.

The primary outcomes of interest were the detection and diagnosis of dementia using dual-task assessments in contrast to standard cognitive or single-task measures. Secondary outcomes included the specific types of dual-task paradigms used, the sensor technologies employed, the quantitative features extracted from assessments, and the analytical methods applied, including statistical and machine learning approaches.

This review considered cross-sectional, case-control, and cohort studies to evaluate the effectiveness and predictive validity of dual-tasking vs. single-tasking in distinguishing cognitive and motor impairments. Only peer-reviewed, full-text journal articles published in English between 2014 and 2025 were included in this review. The focus was maintained on studies providing direct group comparisons to assess whether dual-task paradigms offer greater sensitivity in detecting dementia than single-task approaches.

The exclusion criteria for this systematic review included:

- Studies in which dementia was caused by other underlying conditions such as PD, CSVD, stroke, or MS.
- Studies that did not include a dual-task component (i.e., single-task only or functional tasks without cognitive-motor interaction).
- Studies that did not include a relevant comparator group (e.g., healthy controls or other cognitive groups).
- Studies not reporting extracted features, sensor-derived data, or analytical methods related to dual-task assessments.
- Conference abstracts, reviews, case reports, non-peer-reviewed articles, and interventional or longitudinal studies.

### 3.4 Risk of bias analysis

We used an adapted version of the Newcastle-Ottawa Scale (NOS) to assess methodological quality. The NOS was selected because the included studies were primarily observational (cross-sectional, case-control, or cohort designs) [24, 25]. The scale was tailored to reflect methodological features specific to dual-task dementia detection research which is a well-established practice in systematic reviews [26]. The assessment evaluated four primary domains: clearness of Stated Aim, Sample Selection, Comparability and Outcomes. This included specific evaluations for dementia diagnosis, dual-task protocols, and sensor specifications. The maximum possible score was 16 points. Studies were categorised into three predefined quality tiers based on total scores: 13–16 points indicated high quality (low risk of bias), 9–12 points indicated moderate quality (moderate risk), and 8 points or below indicated low quality (high risk) with a high risk of bias.

### 3.5 Data extraction

All retrieved records were uploaded into Covidence, an online systematic review management platform. The software was used to detect and remove duplicate entries, conduct title and abstract screening, and facilitate the full-text eligibility assessment. Data were independently extracted by two reviewers (I.H and M.G) using a standardised data extraction form. The extracted information included study characteristics (e.g., title, author, year of publication), participant details (e.g., cohort type, sex, age, presence of dementia or related conditions), and assessment methods (e.g., cognitive assessments, single-task and dual-task evaluations). Additionally, we recorded the types of sensors used, analytical approaches (e.g., statistical methods, machine learning algorithms), key findings, extracted features, and diagnostic outcomes.

### 3.6 Data synthesis

We applied a meta-analysis to rigorously evaluate which extracted in the included studies show the largest, most consistent differences between people with dementia and healthy control group. By pooling standardised effect size (Cohen’s d) across multiple studies, we aimed both to order features, from strongest to weakest, by their discriminative power and to assess the precision and reliability of those estimates. We chose inverse-variance weighting because it optimally combines information according to each study’s precision, and we report 95% confidence intervals to convey the uncertainty around each pooled effect.

For each study and feature, we extracted group sample sizes ($n_{\mathrm{dem}}$, $n_{\mathrm{ctrl}}$), means (${\bar{X}}_{\mathrm{dem}}$, ${\bar{X}}_{\mathrm{ctrl}}$), and standard deviations ($S_{\mathrm{dem}}$, $S_{\mathrm{ctrl}}$).

In our quantitative synthesis, we first extracted, for each study and each feature, the sample size ($n_{\mathrm{dem}}$, $n_{\mathrm{ctrl}}$), group means (${\bar{X}}_{\mathrm{dem}}$, ${\bar{X}}_{\mathrm{ctrl}}$) and standard deviations ($S_{\mathrm{dem}}$, $S_{\mathrm{ctrl}}$). We computed Cohen’s d for each comparison as shown in Equation 1,

$$d_{i}=\frac{{\bar{X}}_{\mathrm{dem}}-{\bar{X}}_{\mathrm{ctrl}}}{S_{\mathrm{pooled}}}$$ (1)

where the pooled standard deviation was calculated as shown in Equation 2,

$$S_{\mathrm{pooled}}=\sqrt{\frac{(n_{\mathrm{dem}}-1)S_{\mathrm{dem}}^{2}+(n_{\mathrm{ctrl}}-1)S_{\mathrm{ctrl}}^{2}}{n_{\mathrm{dem}}+n_{\mathrm{ctrl}}-2}}.$$ (2)

The sampling variance of each effect size was then computed using Equation 3,

$$\text{Var}(d_{i})=\frac{n_{\mathrm{dem}}+n_{\mathrm{ctrl}}}{n_{\mathrm{dem}}n_{\mathrm{ctrl}}}+\frac{d_{i}^{2}}{2(n_{\mathrm{dem}}+n_{\mathrm{ctrl}})}$$ (3)

Because several included studies had modest sample sizes, we additionally calculated Hedges’ g as a small-sample corrected version of Cohen’s d as shown in Equation 4:

$$g_{i}=Jd_{i},\;J=1-\frac{3}{4(n_{\mathrm{dem}}+n_{\mathrm{ctrl}}-2)-1}.$$ (4)

The sampling variance of $g_{i}$ was obtained by multiplying the variance of $d_{i}$ by $J^{2}$. Given the expected heterogeneity arising from differences in sensing devices, walking protocols, and dual-task paradigms, a random-effects model was adopted as the primary analytic framework. Between-study variance ($\tau^{2}$) was estimated using restricted maximum likelihood (REML) [27]. Random-effects weights were defined as shown in Equation 5,

$$w_{i}^{*}=\frac{1}{\text{Var}(d_{i})+\tau^{2}},$$ (5)

and pooled effects were calculated as shown in Equation 6,

$$\hat{d}=\frac{\sum_{i}w_{i}^{*}d_{i}}{\sum_{i}w_{i}^{*}}.$$ (6)

Ninety-five percent confidence intervals were computed as $\hat{d}\pm 1.96\;\text{SE}(\hat{d})$, where $\text{SE}(\hat{d})=\sqrt{1/\sum_{i}w_{i}^{*}}$. To characterise the expected dispersion of true effects across heterogeneous study settings, 95% prediction intervals were additionally calculated using Equation 7,

$$\hat{d}\pm 1.96\sqrt{\tau^{2}}.$$ (7)

Statistical heterogeneity was quantified using Cochran’s Q, the inconsistency statistic $I^{2}$, and the between-study variance *τ*^2^ where *I*^2^ was calculated as shown in Equation 8,

$$I^{2}=\max\left(0,\frac{Q-(k-1)}{Q}\right)\times 100\%.$$ (8)

Features reported in fewer than two studies (k<2) were excluded from quantitative synthesis.

To investigate the influence of cognitive loading, we performed subgroup analyses by stratifying data into single-task vs. dual-task, and then, within the dual-task group, into memory and verbal fluency vs. arithmetic cognitive loads. Each subgroup analysis used the same REML framework. Sensitivity analyses were conducted using leave-one-out procedures for each pooled feature. Features exhibiting very high heterogeneity (e.g., $I^{2}>90\%$) were examined separately, and any exclusions or re-analyses were transparently reported to determine whether pooled estimates remained directionally and statistically consistent.

For each subgroup, we ran the same Cohen’s d and inverse-variance pooling pipeline, again retaining only those features with k>1. Sensitivity analyses involved iteratively removing features or subgroups with extreme heterogeneity (e.g., $I^{2}>90\%$) and confirming that pooled estimates remained directionally and statistically consistent. This approach allowed us to identify not only which extracted features differed most robustly between dementia and control groups, but also how cognitive challenge modulated those differences and how stable the findings were to the exclusion of outlying studies or highly variable metrics.

## 4 Results

This section describes the systematic screening and selection procedure undertaken in this review. The findings corresponding to each research question are presented in the subsequent subsections.

### 4.1 Study selection

**Figure 1:**
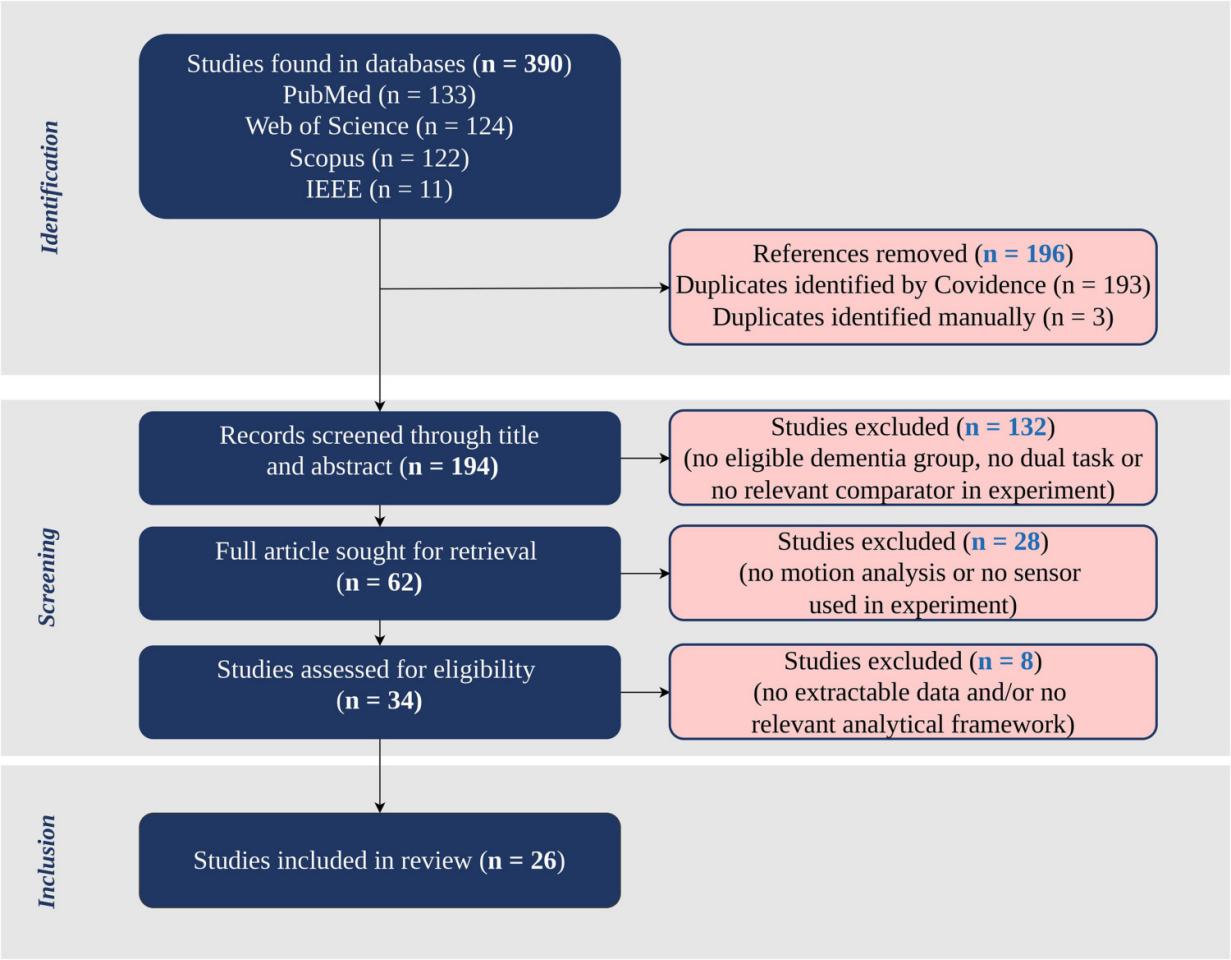
PRISMA flow diagram of the study selection process.

The study identification and selection process is illustrated in Figure 1. The database search yielded a total of 390 records, comprising PubMed (n=133), Web of Science (n=124), Scopus (n=122), and IEEE (n=11). After removing 196 duplicate records (193 identified using Covidence and 3 manually), 194 unique records remained for title and abstract screening. At this stage, 132 records were excluded due to the absence of an eligible dementia cohort, lack of a dual-task paradigm, absence of a relevant comparator group, or failure to meet primary peer-reviewed study criteria. Sixty-two full-text articles were retrieved for further assessment. During full-text evaluation, 28 studies were excluded because they did not include motion analysis or did not employ sensor-based measurement within the experimental design. A further eight studies were excluded due to the absence of extractable quantitative data and/or a relevant analytical framework for dementia-related feature analysis. Ultimately, 26 studies met all predefined inclusion criteria and were included in the final review. The PRISMA flow diagram (Figure 1) provides a transparent overview of the identification, screening, eligibility assessment, and inclusion stages. A structured summary of the included studies is presented in Supplementary Table S1, detailing participant cohorts, cognitive assessments, experimental paradigms, sensor modalities, extracted features, and analytical approaches. These methodological characteristics form the basis of the structured synthesis presented in the subsequent sections.

A total of 26 studies met the inclusion criteria and were included in this review. To address the research questions outlined in this review, the extracted data from the included studies were categorised into several key themes. These themes correspond to core components of dual-task assessment and align with the aims of understanding the types of tasks used, the assessment mechanisms, and the methods employed to interpret the results. It begins with an overview of participant cohorts and cognitive assessments used across the included studies. This provides important context regarding the diagnostic categories and cognitive status applied, which lays the foundation for interpreting differences observed in dual-task performance and supports the analysis presented in the subsequent sections.

The following categories address each research question directly. Experimental paradigms are explored to answer RQ1 by identifying the types of dual-task paradigms used and how task combinations and prioritisation strategies are managed. Measurement approaches, including wearable sensor technologies, are examined in relation to RQ2, focusing on how gait, balance, and cognitive performance are assessed and what quantitative features are extracted. Finally, analytical methods and mechanisms are reviewed to address RQ3, highlighting the statistical and machine learning techniques used to interpret dual-task data and supporting RQ4 by identifying the most promising tasks and features for dementia detection.

In order to focus our quantitative synthesis on the task and features and directly answer RQ4, we performed meta analyses to those studies that shared similarities in the primary motor function and their extracted features. In this case, walking test was selected and included in the analysis. Accordingly, we extracted, for each eligible study, the means, standard deviations, and sample sizes for dementia and control groups on every gait and turning metric. We then computed Cohen’s d and its variance for each comparison and applied inverse-variance weighting to obtain a precision-weighted pooled effect size per feature. Ninety-five percent confidence intervals around these pooled estimates quantify their statistical certainty, and any metric with fewer than two contributing studies was excluded to preserve robustness. Finally, we repeated this procedure separately for single-task walking and for dual-task conditions, stratifying dual-task into memory and verbal fluency and arithmetic loads, so that we could directly compare how each cognitive challenge modulates the discriminative power of the retained walking features.

### 4.2 Methodological quality and risk of bias

**Figure 2:**
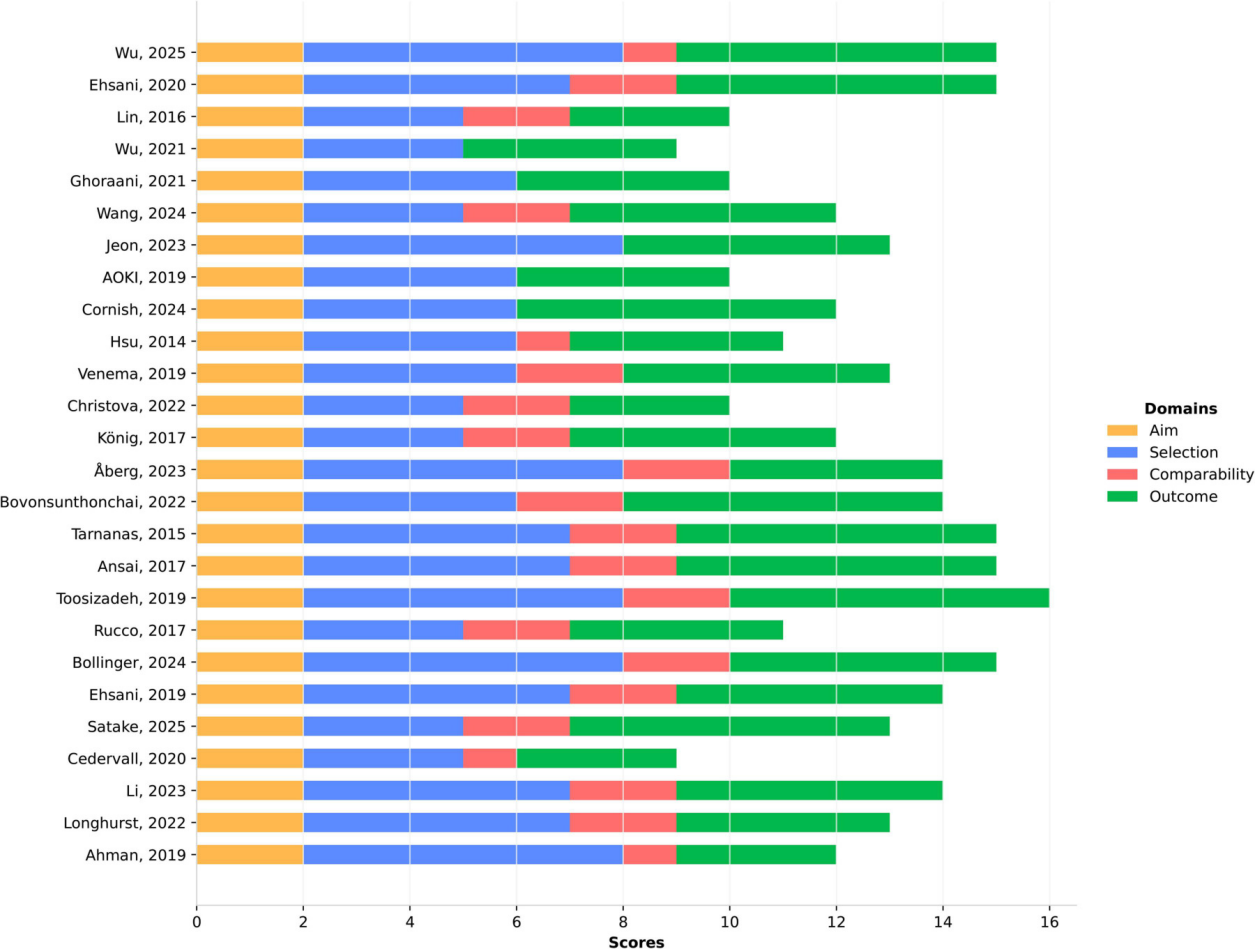
Methodological quality and risk of bias assessment of the included studies using the adapted Newcastle-Ottawa Scale. Stacked bars indicate the score breakdown across the four evaluated domains (Aim, Selection, Comparability, and Outcome) for each study, with a maximum possible score of 16.

Figure 2 illustrates the risk of bias assessment for the 26 included studies using the adapted cross-sectional Newcastle-Ottawa Scale. Total quality scores ranged from 9 to 16 out of a possible 16 points. Overall, 14 studies (54%) demonstrated high methodological quality, and 12 studies (46%) achieved a moderate quality rating. No studies were classified as low quality.

### 4.3 Participant cohorts

**Figure 3:**
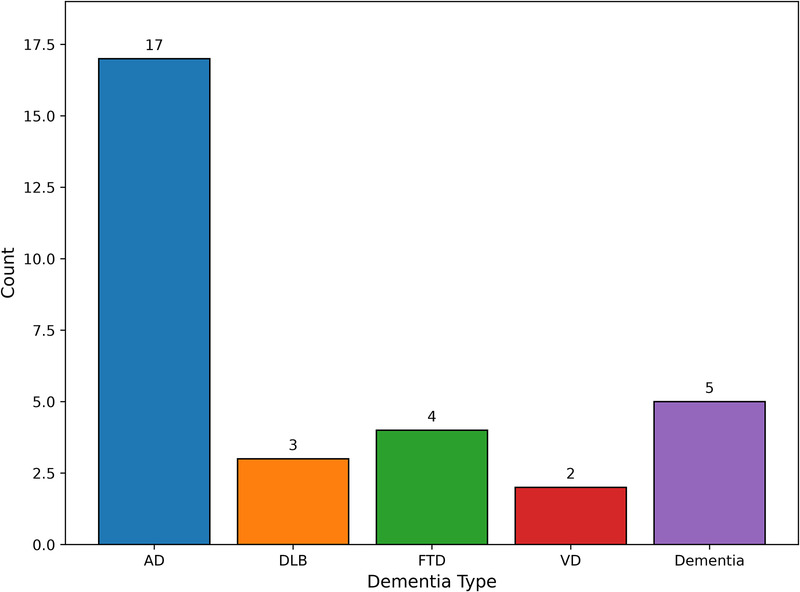
Distribution of single motor task assessments across the reviewed studies.

The studies included in this systematic review encompassed a range of dementia types, reflecting the heterogeneity of cognitive decline observed in older adults. As illustrated in Figure 3, AD was by far the most frequently investigated condition, featured in 17 studies. In contrast, other dementia types were considerably less represented. FTD was examined in four studies, VD in two, and DLB in three studies. Additionally, five studies referred more broadly to dementia without specifying subtypes, indicating a focus on general cognitive impairment rather than a clinically distinct diagnosis.

Several studies clearly delineated participant cohorts into cognitively impaired and non-impaired groups using standardised cognitive assessments including Montreal Cognitive Assessment (MoCA) [28] and Mini-Mental State Examination (MMSE) [29]. In the included studies, cognitive screening tools were typically used to assess cognitive status or severity of impairment rather than to establish the clinical diagnosis of dementia subtype. Five studies specifically used cognitive screening tools to categorise their participants into high and low cognitive function groups [30–34]. One such study classified participants into two groups based on MoCA scores: 27 participants with high cognition (MoCA ≥ 26) and 23 participants with low cognition (MoCA < 26) [31]. These cognitive assessments were not only used to define inclusion criteria but also served as tools for stratifying participants in analyses and subgroup comparisons.

The most commonly used comparison group consisted of healthy older adults with normal cognition (NC). These control groups were generally used to contrast the dual-task performance against those diagnosed with dementia. For example, in studies that included participants with dementia and/or MCI, the control groups were often matched in age and the number of participants but did not exhibit signs of cognitive decline [35–38]. This approach helps enhance the between-group analyses and improve the reliability of identifying significant differences in dual-task performance.

### 4.4 Experimental paradigms

This section provides an overview of the motor function and cognitive assessment methods used across the included studies. In line with RQ2, we categorised the experimental protocols into three groups: single motor task, single cognitive task, and dual-task paradigms. All of the 24 included studies implemented both single and dual-task conditions, sharing the same primary motor task across conditions to allow for direct comparison. Identifying the nature of this primary motor task is essential, as it forms the foundation upon which cognitive interference and dual-task cost are evaluated. The assessments primarily focus on gait and/or balance tasks, which are then paired with cognitive activities in dual-task settings to examine their impact on performance and their utility in detecting cognitive decline.

#### 4.4.1 Single tasks

**Figure 4:**
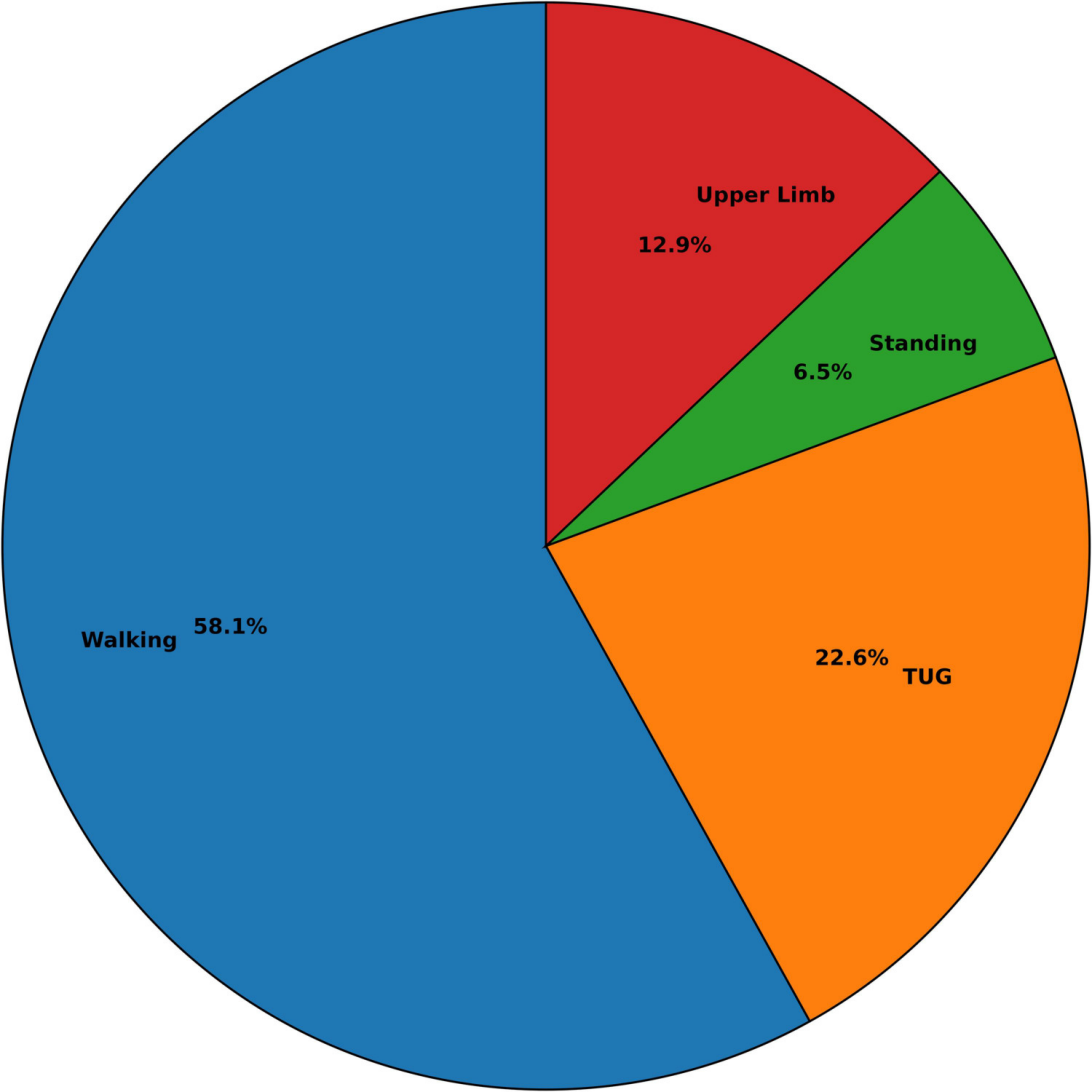
Distribution of single motor task assessments across the reviewed studies.

Single-task protocols involved a physical activity administered in isolation prior to introducing a secondary physical or cognitive load. These tasks primarily aimed to evaluate baseline motor without the added complexity of multitasking. As shown in Figure 4, walking-based assessments were the most common (n=18), followed by the Timed Up and Go (TUG) test (n=7), standing balance tasks (n=2), and upper-limb motor assessments such as arm/elbow flexion (n=4). A few studies also incorporated multiple single motor tasks within the same protocol, such as walking combined with TUG [31, 39], walking and balance tests [40] or walking and upper limb test [34, 41] to examine different aspects of motor performance across varying levels of complexity. In one study by Jeon et al. [42], in addition to normal walking, participants were asked to perform walking with obstacle crossing, where a large physical barrier required subjects to visually perceive and navigate around it adding a level of motor planning and visual-spatial demand to the otherwise single walking task.

Walking tasks typically involved overground or treadmill walking across a fixed distance and were used to capture steady-state gait parameters. The TUG test, which incorporates standing, walking, turning, and sitting transitions, provided insight into dynamic balance and functional mobility. These single-task assessments enabled the extraction of a wide range of spatiotemporal and postural features, such as gait speed, stride length and variability, cadence, and double support time. Standing tasks commonly analysed postural sway and centre of pressure movement to assess balance control. Upper-limb tasks, such as arm flexion, were used less frequently and typically aimed at evaluating motor coordination and movement control, while arithmetic-based cognitive tasks assessed working memory and cognitive processing speed under low motor demand.

While all studies employed a single motor task, five studies included single cognitive tasks, administered separately, to establish a baseline measure of cognitive performance before introducing dual-task conditions. These tasks, such as serial subtraction, verbal fluency, or word recall, were performed in a sitting position to assess cognitive function without the confounding influence of a concurrent physical activity [31, 32, 43]. This approach helped isolate key cognitive domains such as attention, working memory, and processing speed. For instance, Longhurst et al. [43] found that dual-task conditions significantly increased the time required for serial subtraction tasks. Wu et al. [32] reported that calculation speeds declined notably under dual-task conditions. By evaluating both motor and cognitive tasks independently, studies were able to better interpret performance changes during dual-tasking and determine whether impairments were due to cognitive load alone or the interaction between cognitive and motor demands.

#### 4.4.2 Dual tasks

**Figure 5:**
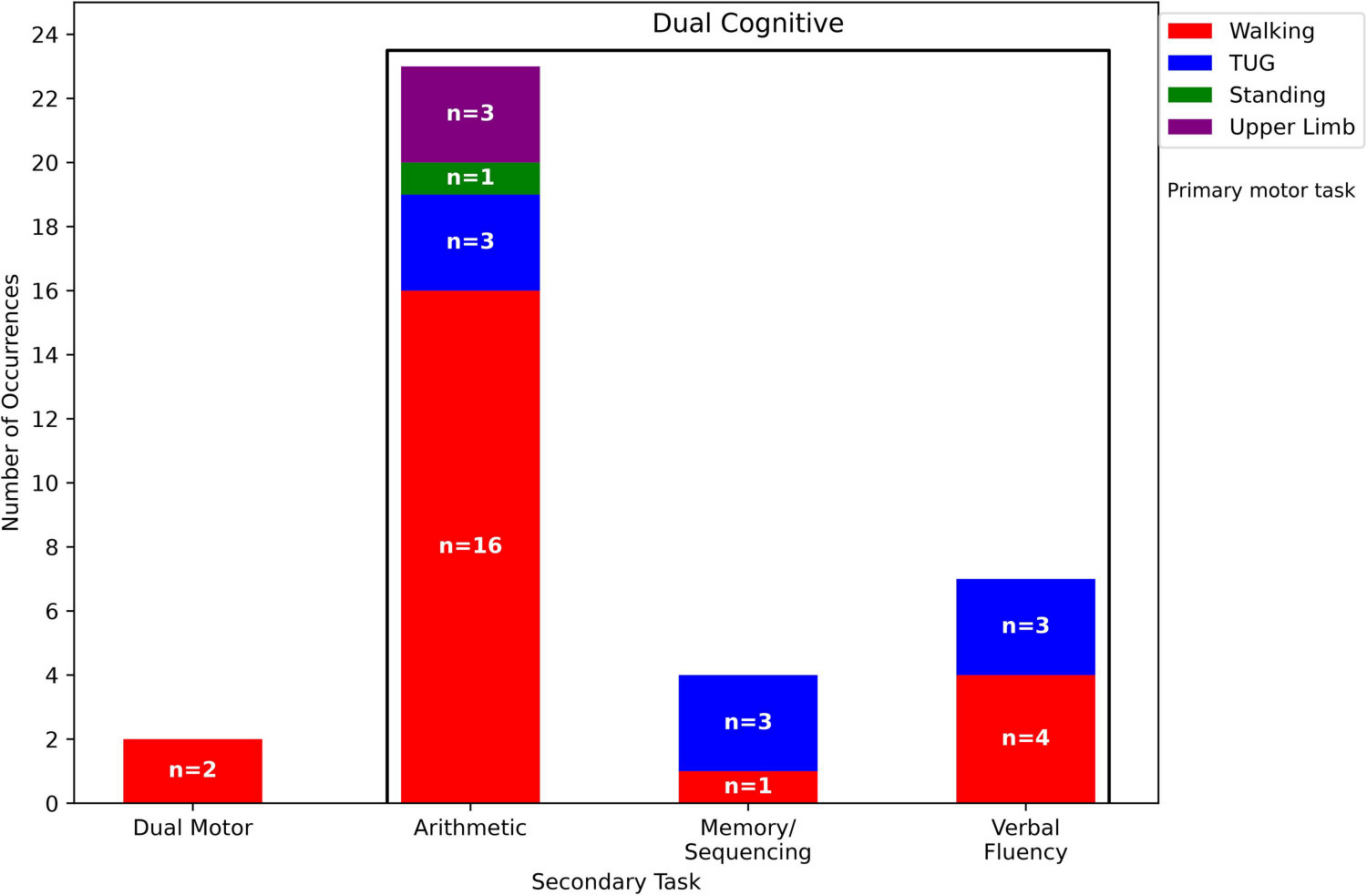
Distribution of secondary tasks along with their corresponding primary tasks in dual-task assessment paradigms across reviewed studies.

In dual-task paradigms, a secondary task, either cognitive or motor, is introduced alongside a primary motor task (e.g., walking or TUG) to simulate real-life multitasking demands and assess cognitive-motor interference. The secondary task is designed to impose an additional load on attentional or executive resources, thereby allowing researchers to measure the performance decrement (i.e., dual-task cost) compared to baseline single-task conditions. This approach helps reveal subtle impairments in coordination, attention allocation, and task prioritisation that are often characteristic of early-stage dementia. Figure 5 presents the distribution of dual-task assessments used in the studies, categorised by cognitive or motor task type and further stratified by the primary motor task with which they were paired. Cognitive secondary tasks were categorised according to their dominant cognitive demand into arithmetic tasks, verbal fluency, and memory or sequencing tasks, following common classifications used in dual-task gait research [44]

As illustrated in Figure 5, dual cognitive tasks were far more prevalent than dual motor tasks across the reviewed studies, representing 24 cases compared to only 2 cases, respectively. Among these, the most commonly used paradigm was an arithmetic-based cognitive task performed concurrently with a walking task, reported in 16 studies. In addition, arithmetic tasks were also paired with the TUG [31, 43, 45] and upper limb tasks [34, 46, 47] test in three studies each, with standing in one study [48]. The secondary arithmetic tasks varied in complexity across studies. Some employed relatively simple tasks such as counting forward by one [31] or backward by one [30, 31, 34, 35, 37, 40, 42, 46, 47, 49, 50] from a predefined number, which required basic attention and sequencing skills. Other studies increased the cognitive challenge by using subtraction tasks with greater intervals, such as counting forward by three [41], counting backward by three [31, 33, 34, 41, 43, 46–48, 51], and counting backward by seven [36, 45, 48, 50] from a specified starting number. These more demanding tasks were intended to impose higher cognitive load, engaging working memory, and executive function more intensively. For example, in a study by Venema et al. [31], participants performed walking and TUG tasks while engaging in cognitive tasks of varying difficulty, such as counting forward by ones and counting backward by threes from a randomised starting number; the counting backward-by-three task was shown to impose a significantly greater cognitive load, which resulted in a higher dual-task cost. Two studies implemented dual-task paradigms in which participants performed a walking task while solving arithmetic problems involving addition and subtraction of one- and two-digit numbers, with questions generated to maintain sustained cognitive load [52, 53]. In another study by Wu et al. [32], participants engaged in a walking task while simultaneously solving arithmetic problems of varying difficulty levels, ranging from simple addition of single-digit numbers to more complex subtraction involving two-digit numbers, over a thirty-second trial. These randomly generated questions ensured variability and helped impose a sustained cognitive load, making the task suitable for detecting lower MMSE scores. Verbal fluency and memory/sequencing cognitive tasks were used less frequently, primarily in combination with TUG (n=4) and walking (n=5). Verbal fluency tasks typically required participants to generate words within a semantic category while performing the motor task. For example, several studies instructed participants to name as many animals as possible within a fixed time window [38, 49, 50, 54–56]. In another study, participants generated semantically related words from predefined categories such as animals, plants, fruits, food, and colors while walking [42]. These tasks primarily engage lexical access, semantic retrieval, and executive control.

Verbal fluency and memory or sequencing cognitive tasks were used less frequently, primarily in combination with TUG (n=6) and walking (n=5). Verbal fluency tasks required participants to generate words within a semantic category while performing the motor task. For example, several studies instructed participants to name as many animals as possible within a fixed time window [38, 49, 50, 54–56]. In another study, participants generated semantically related words from predefined categories such as animals, plants, fruits, food, and colors while walking [42]. These tasks primarily engage semantic retrieval, lexical access, and executive control. Memory or sequencing tasks required participants to recall or manipulate ordered information while maintaining gait performance. For example, participants were asked to recite the alphabet [51] or state the months of the year in reverse order [54, 55], which involve maintaining and manipulating sequential information in working memory. One study also incorporated an explicit memory recall task in which participants with AD or MCI were required to recall and state a phone number while walking [39]. Compared with arithmetic tasks, these tasks typically involve broader cognitive processing but place less structured demand on numerical manipulation, engaging semantic retrieval, working memory, and attentional control during dual-task walking.

Additionally, two studies implemented a motor dual-task assessment alongside a dual cognitive task. In one study, participants were instructed to walk at a normal pace while carrying a tray with two glasses filled with water. The spatio-temporal parameters related to velocity and stability were captured using a stereophotogrammetric system with eight infrared cameras [36]. Another study introduced a complex dual-task condition in which participants performed walking and simultaneous chip-swapping in both hands while engaging in one of three secondary tasks: speaking associative words, counting numbers in ascending order, or counting in descending order [42]. This multi-tiered design was used to evaluate walking ability, motor coordination, memory, and cognitive function under high cognitive-motor load.

### 4.5 Measurement devices

**Figure 6:**
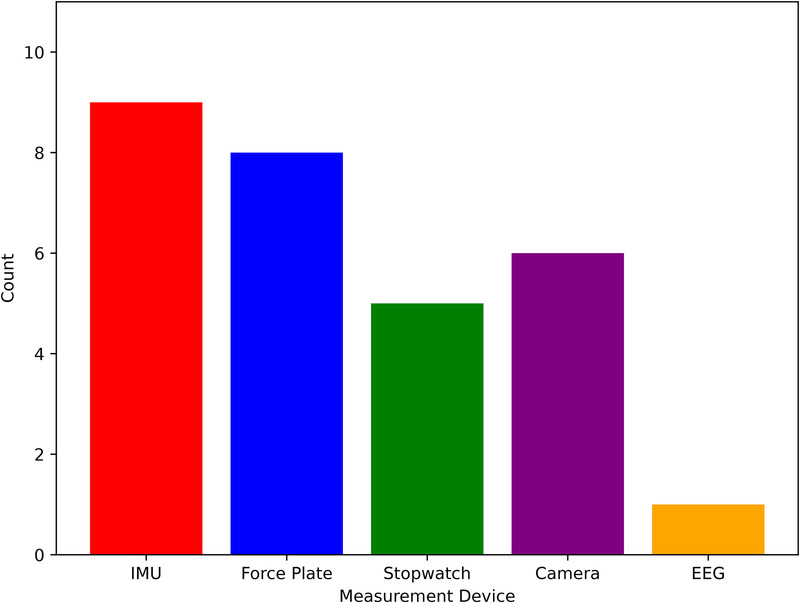
Measurement techniques used across the reviewed studies.

The reviewed studies employed a variety of measurement techniques to measure and analyse participants’ motor during single and dual-task assessments. These technologies can be broadly categorised into wearable sensors, which are attached directly to the body, non-wearable sensors, which involve externally placed equipment such as force plates, cameras, and timing tools. The distribution of measurement approaches used in the studies is presented in Figure 6.

#### 4.5.1 Wearable sensors

Wearable sensors were among the most commonly used technologies in the reviewed studies, offering flexibility and ecological validity for both lab-based and real-world assessments. The most frequently employed wearable devices were IMUs, used in 9 studies [34, 35, 38, 40, 42, 45–47, 50]. IMUs, which typically consist of accelerometers and gyroscopes, were often affixed to key body locations such as the lower back, thighs, ankles, or feet to capture spatiotemporal gait parameters. These included gait speed, stride length and variability, step time, and turn characteristics. Their unobtrusive nature and capacity for high-frequency data collection made them ideal for both overground and treadmill walking tasks under single or dual-task conditions. In addition to biomechanical sensing, one study incorporated electroencephalography (EEG), integrating patch-type EEG signals with dual-task measures in a multi-modality fusion framework for dementia detection [53]. This approach combined neural activity with gait and cognitive features within the same assessment paradigm.

#### 4.5.2 Non-wearable sensors

Non-wearable sensor systems were also widely used, particularly in controlled laboratory settings. Force plates, also used in 8 studies, were primarily employed during walking [31, 32, 37, 41, 49, 51], standing balance tests [48] or during phases of the TUG test [31] that involved transitions such as sit-to-stand or turning. These platforms provided precise measurements of centre of pressure, sway velocity, and balance control metrics, which offer insight into postural stability of the participants in different cohorts.

In addition, six studies employed camera systems for gait analysis. Two studies used Qualisys systems with seven or eight infrared cameras to capture 3D kinematic data and analyse gait stability, joint angles, and transitional movements during tasks such as TUG and dual-task walking [36, 39]. One study used a Vicon MX system in conjunction with force plates to evaluate both lower-limb gait parameters and upper-limb motor responses during dual-task conditions [41]. Three studies implemented the Microsoft Kinect V2 camera, a markerless infrared depth sensor, to track stepping performance and joint movements during an automated dual-task system [32, 52, 53]. These camera systems enabled detailed analysis of step patterns, joint excursions, and movement coordination, offering valuable insights into motor-cognitive interaction in older adults.

#### 4.5.3 Timing tools

Stopwatches were used in five studies to record the total time taken to complete TUG test [33, 43, 54–56]. Although less precise than automated sensors, stopwatches have served as a practical and accessible tool in clinical or low-resource environments.

### 4.6 Extracted features

Across the reviewed studies, a wide range of features were extracted from the raw data collected during motor and cognitive assessments. These features were intended to capture clinically meaningful patterns relevant to mobility, postural control, upper limb movement, cognitive functioning, and task interference. Most studies computed spatio-temporal parameters from walking or TUG assessments, balance features from static stance tasks, and various kinematic or entropy-based features from upper limb movements. In dual-task scenarios, features were often designed to quantify performance decrements relative to single-task conditions. Extraction techniques varied across studies, with some relying on straightforward time-domain statistics, while others employed more advanced frequency-domain or nonlinear dynamic analyses. This section provides an overview of the types of features extracted in each functional domain and outlines how they were derived from the recorded data.

#### 4.6.1 Gait parameters

Gait assessments varied considerably across the reviewed studies, even among those using similar sensor modalities. As shown in Supplementary Table S1, thirteen studies reported quantitative gait features, most commonly extracted during walking or TUG test. The most frequently reported parameters were stride time (n=7), cadence (n=8), stride length (n=7), swing time (n=4), step length (n=5), gait speed (n=6), and step time (n=4). Other reported metrics included gait variability (n=2), stride width (n=2), and step regularity (n=1). Several studies also considered composite features such as stride-to-stride variability and swing period asymmetry to better capture instability or irregular gait patterns. This section focuses on the most commonly reported features—those mentioned in two or more studies, including stride time, stride length, cadence, and gait speed, due to their established relevance in identifying motor and cognitive decline.

Stride time was one of the most commonly extracted gait features across multiple studies and served as a critical indicator of gait and cognitive-motor integration [31, 37, 40–42, 50, 51]. Bovonsunthonchai et al. [37] stated that individuals with mild AD exhibited significantly prolonged stride time compared to both MCI and NC participants under dual-task conditions. This increase in stride time was more pronounced when participants performed cognitive tasks while walking, highlighting the sensitivity of this feature to attentional load and executive dysfunction. Similarly, Ghoraani et al. [51] found that stride time and its variability were among the most significant gait features for classifying healthy, MCI, and AD groups. They extracted stride time metrics across multiple trials and used machine learning to demonstrate that dual-task stride time, especially its standard deviation and dual-task cost, was highly effective in distinguishing AD from NC groups. Although different studies used various task conditions (e.g., single-task vs. dual-task) and gait measurement systems, they consistently identified increased stride time, and particularly its variability, as a hallmark of cognitive impairment progression. This convergence highlights stride time as a reliable and sensitive parameter for differentiating among healthy, MCI, and dementia populations.

Cadence, typically defined as the number of steps per minute, was a frequently extracted gait feature across several reviewed studies and demonstrated a consistent sensitivity to cognitive impairment, particularly under dual-task conditions [31, 35–38, 41, 42, 51]. In the study by Bovonsunthonchai et al. [37], cadence significantly decreased in participants with dementia compared to both MCI and NC groups during both single and dual-task walking, with dual-tasking further exacerbating the group differences. Similarly, König et al. [35] found that cadence was reduced in AD and MCI groups during dual-task walking compared to NC group, although no significant difference was observed between MCI and NC under single-task conditions. This pattern supports the hypothesis that cognitive load strongly impacts motor performance in cognitively impaired individuals. Rucco et al. [36] also reported a notable decline in cadence in both AD and frontotemporal dementia groups during cognitive dual-tasking, with the AD group exhibiting a significant reduction in cadence compared to NC group. The convergence of these results across studies suggests that cadence is a sensitive marker of cognitive decline, especially when executive function demands are elevated. However, while the overall trend points toward a decline in cadence in dementia populations, the extent of change and its discriminative power between MCI and NC groups appeared less consistent, indicating that cadence may be more robust in identifying more advanced stages of cognitive impairment.

Stride length was one of the most frequently reported gait features across the reviewed studies, particularly in dual-task contexts [31, 36–38, 41, 45, 51]. Consistent findings emerged in multiple papers, which demonstrated that individuals with cognitive impairment, especially those with AD, tend to exhibit reduced stride length when compared to NC group. For instance, in the study by Rucco et al. [36], stride length significantly decreased in AD patients during both single and dual-task conditions, with further impairments observed under cognitive dual-tasking. The authors noted that stride length reduction in AD occurred alongside alterations in kinematic patterns and increased stride length variability, suggesting compromised motor control under executive function load. Similarly, Bovonsunthonchai et al. found that individuals with mild AD had significantly shorter stride lengths compared to controls, particularly under dual-task conditions that required backward counting. The group differences were statistically significant and accentuated under increased cognitive demand. In another study, Li et al. [45] reported that stride length during both single- and dual-task walking was significantly reduced in the AD group (mean ST = 81.61 cm; DT = 70.99 cm) compared to both MCI and NC groups. This reduction aligned with decreased gait velocity and suggested broader disruptions in gait efficiency. In contrast, Venema et al. [31] provided a more nuanced view, where dual-task cost in stride length was significantly more negative in the cognitively impaired group compared to NC group. This suggests that while stride length alone may not always distinguish between early cognitive states, its response to cognitive load, especially through dual-task paradigms, can enhance its discriminative power. Overall, stride length is a reliable marker for cognitive impairment, particularly when assessed during dual-tasking. However, its sensitivity to MCI varies depending on study design, task complexity, and the stage of cognitive impairment.

Swing time, a key component of the gait cycle representing the duration the foot is in the air during walking, emerges as an informative parameter for differentiating cognitive conditions in the context of dual-task gait assessments [36, 40, 42, 51]. Across multiple studies, changes in swing time—either in absolute values or variability—were often associated with dementia. For instance, Hsu et al. [40] reported that swing time was longer in AD patients compared to NCs during dual-task walking, though the difference was not significant during single-task conditions. Importantly, they found a significantly greater coefficient of variation (CV) in swing time in AD patients under dual-task conditions, suggesting impaired gait stability when cognitive demands increases. In a study employing 3D motion capture, Rucco et al. [36] also found that swing time was increased in patients with behavioural variant frontotemporal dementia and, to a lesser extent, in AD patients, particularly during dual-task walking. Moreover, they observed reduced angular joint motion during swing (notably knee and thigh excursion), which correlates with altered or prolonged swing phases. This aligns with findings that cognitive load, especially from tasks taxing executive function, interferes with the timing and control of gait phases. Interestingly, while swing time was prolonged under cognitive challenge in the AD group in both studies, the magnitude and significance varied, possibly due to differences in task design, gait measurement methods (e.g., IMU sensors vs. camera systems), or dementia stage.

Step length has been explored across several studies in the context of cognitive impairment and dementia, and consistent findings have emerged from multiple sources [31, 37, 51, 55, 56]. Bovonsunthonchai et al. [37] found that step length significantly differed among NC individuals, those with MCI, and those with dementia, with progressively shorter steps observed as cognitive function declined. These differences were especially notable under dual-task conditions. Similarly, Cedervall et al. [56] used a dual-task version of the TUG test and found that individuals with dementia had significantly shorter step lengths after turning compared to those without a dementia diagnosis.

Gait speed is a commonly analysed feature in studies assessing cognitive impairment, often serving as an accessible and non-invasive marker for detection of dementia [31, 37, 38, 45, 50, 51]. Several of the reviewed studies confirm its diagnostic value, particularly under dual-task conditions. Li et al. [45] observed significantly slower gait speeds in individuals with AD and MCI compared to NC individuals during both single-task and dual-task walking. The study found that gait speed (mean stride velocity) was substantially reduced in the AD group and moderately reduced in the MCI group relative to NCs, especially under dual-task conditions, which aligns with the notion that dual-task gait tasks are more cognitively demanding and thus more sensitive to early cognitive decline. Similarly, in the study by Ghoraani et al. [51], gait speed was significantly lower in participants with MCI and AD compared to NC group across both single and dual-task conditions. Notably, this study emphasised the role of dual-task gait speed as a critical parameter in distinguishing between groups. In another study, Venema et al. [31] reinforced this perspective by analysing the dual-task cost of gait speed and found that individuals with cognitive impairment experienced significantly higher dual-task cost compared to NC group. This suggests that cognitive load disproportionately affects motor function in impaired groups and that the reduction in gait speed under dual-tasking reflects executive dysfunction.

**Figure 7:**
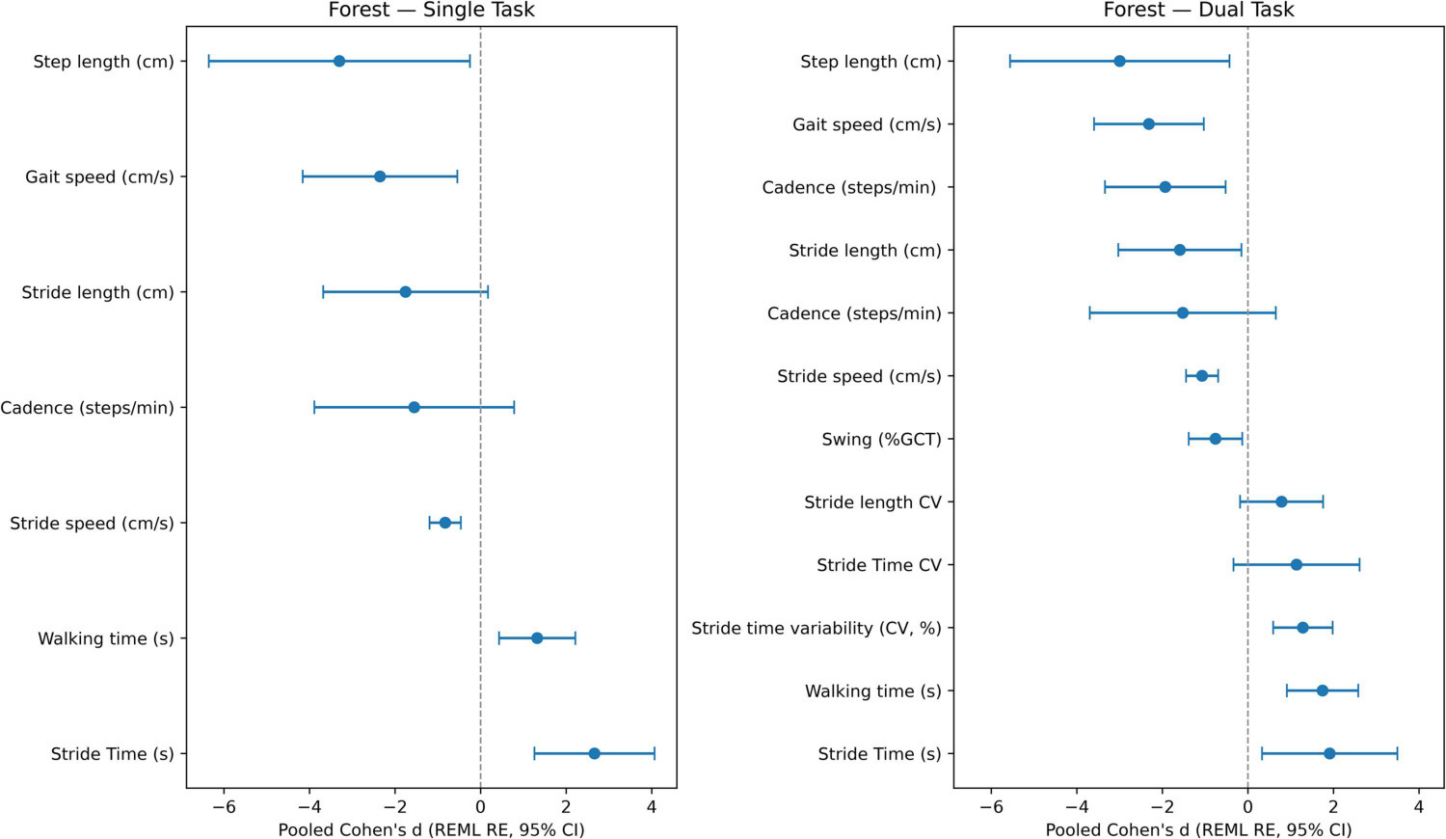
Random-effects (REML) pooled Cohen’s d (95% CI) for gait outcomes distinguishing dementia from controls under single- and dual-task conditions.

Figure 7 summarises the random-effects (REML) meta-analysis of single- and dual-task gait outcomes across the ten studies. For each feature, we report the pooled effect size, 95% confidence interval (CI), between-study variance ($\tau^{2}$), heterogeneity ($I^{2}$), and 95% prediction interval (PI). Under single-task gait, step length showed the largest pooled effect (d=−3.30, 95% CI [−6.35,−0.25]), with substantial heterogeneity ($I^{2}=95.2\%$, $\tau^{2}=6.97$). The corresponding prediction interval was wide ([−8.48,1.87]), indicating considerable variation in effect magnitude across studies. Stride time demonstrated a large pooled effect (d=2.66, 95% CI [1.26,4.07], $I^{2}=78.3\%$, $\tau^{2}=0.81$; PI [0.90,4.42]), suggesting prolonged gait cycles in dementia. Walking time also showed a large pooled effect (d=1.32, 95% CI [0.43,2.21], $I^{2}=92.7\%$, $\tau^{2}=0.73$; PI [−0.35,2.99]). Gait speed was significantly reduced (d=−2.35, 95% CI [−4.16,−0.55], $I^{2}=94.9\%$, $\tau^{2}=3.23$; PI [−5.87,1.17]). Other features (e.g., stride length and cadence) exhibited similarly high heterogeneity ($I^{2}>95\%$), with prediction intervals spanning both sides of zero.

Under dual-task gait, large pooled effects persisted. Step length remained strongly reduced (d=−3.00, 95% CI [−5.56,−0.43], $I^{2}=94.3\%$, $\tau^{2}=4.86$; PI [−7.32,1.33]). Gait speed showed a substantial decrease (d=−2.31, 95% CI [−3.60,−1.03], $I^{2}=90.8\%$, $\tau^{2}=2.40$; PI [−5.35,0.72]). Walking time (d=1.74, 95% CI [0.91,2.58], $I^{2}=92.3\%$, $\tau^{2}=0.82$; PI [−0.03,3.51]) and stride time (d=1.91, 95% CI [0.33,3.49], $I^{2}=90.4\%$, $\tau^{2}=1.74$; PI [−0.68,4.50]) were also elevated. Variability measures such as stride time variability (d=1.28, 95% CI [0.59,1.98], $I^{2}=87.9\%$, $\tau^{2}=0.22$; PI [0.36,2.21]) demonstrated increased inconsistency under cognitive load.

Small-sample corrected effect sizes (Hedges’ g) were additionally computed using the same REML framework. Under single-task walking, step length remained the most discriminative feature (g=−3.23), followed by stride time (g=2.59), gait speed (g=−2.31), and walking time (g=1.30), closely mirroring the corresponding Cohen’s d estimates (d=−3.30, 2.66, −2.35, and 1.32, respectively). In dual-task conditions, step length (g=−2.93), gait speed (g=−2.27), stride time (g=1.86), and walking time (g=1.72) likewise showed nearly identical magnitudes to the Cohen’s d values (d=−3.00, −2.31, 1.91, and 1.74). Across all primary outcomes, the absolute difference between Cohen’s d and Hedges’ g was small (maximum Δ≈0.07), corresponding to relative differences of approximately 2 to 3% for the largest pooled effects. The ranking, direction, statistical significance, heterogeneity estimates, and prediction intervals were unchanged. These findings indicate that small-sample bias had minimal impact on the random-effects pooled estimates.

Under arithmetic-based dual-tasking, the largest pooled impairment was observed for gait speed (REML d=−2.75, 95% CI [−4.67,−0.84], $I^{2}=93.7\%$, $\tau^{2}=3.60$; PI [−6.47,0.97]), indicating a marked reduction in forward velocity in dementia. Temporal measures were also strongly affected, with stride time increased (REML d=1.91, 95% CI [0.33,3.49], $I^{2}=90.4\%$, $\tau^{2}=1.74$; PI [−0.68,4.50]). Walking time remained slower under arithmetic load (REML d=0.97, 95% CI [0.57,1.38]), with negligible heterogeneity ($I^{2}=0\%$, $\tau^{2}\approx 0$), suggesting a consistent increase in overall duration across studies. In contrast, verbal fluency dual-tasking accentuated overall gait slowing most strongly. Walking time showed the largest pooled effect (REML d=2.30, 95% CI [1.43,3.18], $I^{2}=78.7\%$, $\tau^{2}=0.51$; PI [0.90,3.70]), indicating substantially prolonged completion time in dementia. Spatial parameters were also impaired, with reduced step length (REML d=−1.68, 95% CI [−2.37,−0.99]) and reduced gait speed (REML d=−1.48, 95% CI [−1.88,−1.07]). For step length, heterogeneity was negligible ($I^{2}=0\%$, $\tau^{2}\approx 0$), whereas gait speed showed modest between-study variability ($I^{2}=44.8\%$, $\tau^{2}=0.04$).

Across most features, heterogeneity was substantial ($I^{2}$ frequently larger than 90%), consistent with differences in devices, gait protocols, and dual-task paradigms. The wide prediction intervals indicate that while the average effect size is large, its magnitude varies considerably across settings. Importantly, however, the direction of effects was generally consistent across studies, supporting the robustness of dementia-related gait impairments. While single-task gait measures capture overall performance, the largest and most clinically informative effects tended to emerge under dual-task conditions. Introducing cognitive load altered the gait profile. That is, arithmetic tasks were associated with pronounced reductions in gait speed and cadence and increased temporal variability, whereas verbal fluency tasks more strongly amplified overall walking duration. Despite substantial between-study heterogeneity, the direction and magnitude of these pooled effects consistently indicated greater cognitive–motor interference in dementia. These distinctions highlight the value of task-specific gait metrics and support the use of dual-task paradigms to enhance sensitivity and specificity of sensor-based biomarkers for dementia screening.

#### 4.6.2 Balance-related features

Only two studies extracted quantitative balance-related features. The most commonly reported parameters were sway path length, sway magnitude, and sway velocity, derived from centre of pressure or acceleration data during static stance tasks. Bollinger et al. [48] quantified postural sway using total sway path length and found significantly higher dual-task cost in sway path length among participants with preclinical AD compared to controls (mean difference 19.8%, p=0.024), indicating greater cognitive-motor interference. Similarly, Hsu et al. [40] extracted sway velocity, root mean square (RMS) acceleration, and displacement range from IMU sensor data, reporting increased sway velocity and RMS acceleration in individuals with AD compared to healthy controls, suggesting these features effectively discriminate balance impairments in AD.

#### 4.6.3 Upper limb-related features

Three studies employed upper limb assessments, particularly using repetitive or rhythmic tasks such as elbow flexion. In the study by Ehsani et al. [46], upper limb performance was evaluated under dual-task conditions, and key extracted features included the number of flexion movements, flexion variability, flexibility variability, entropy, and local dynamic stability. Among these, entropy of angular velocity demonstrated the strongest discriminatory power across cognitive groups. It was significantly higher in individuals with amnestic MCI and early AD compared to NC, suggesting greater movement unpredictability. Additionally, flexion variability and flexibility variability were elevated in the cognitively impaired groups, reflecting less consistent motor execution during dual-tasking. In the study by Ehsani et al. [34], rapid elbow flexion was performed under dual-task conditions, and increased variability in speed and range of motion during upper limb movement emerged as sensitive indicators of cognitive impairment, especially when paired with the more challenging cognitive task of counting backward by threes. Toosizadeh et al. [47] implemented a dual-task elbow flexion test under normal and rapid conditions, showing that flexion number and motion variability could robustly predict early AD and MCI.

### 4.7 Analytical methods and mechanisms

A wide range of analytical strategies was employed across the reviewed studies to interpret dual-task performance and its association with cognitive impairment. These approaches varied in complexity and purpose, from traditional hypothesis-driven statistical testing to more advanced data-driven machine learning techniques aimed at classification and feature selection. To provide a clearer understanding of how dual-task data have been leveraged for cognitive assessment, the analytical methods are discussed under three main categories: statistical analysis, machine learning and combined analysis.

#### 4.7.1 Statistical analysis

Most of the studies (n=13) relied solely on statistical methods to evaluate group differences, quantify dual-task costs, and examine associations between gait or cognitive features and diagnostic categories. For instance, Longhurst et al. [43] applied reliability and validity testing of a dual-task effect battery using repeated-measure Analysis of Variances (ANOVAs) and correlation analyses to compare AD, PD and NC. Lin et al. [41] used mixed-design repeated-measures ANOVAs to examine differences in lower and upper extremity motor performance between AD patients and controls under dual-task conditions. Hsu et al. [40] relied on ANOVA and t-tests to compare gait and balance parameters obtained from inertial sensors between AD and NC during single and dual-task walking. Similarly, Cornish et al. [50] applied repeated-measures ANOVA, paired comparisons, and Bland–Altman analyses to assess agreement between manually counted and algorithm-detected strides across varying gait speeds and single- and dual-task walking conditions.

#### 4.7.2 Machine learning

A smaller subset of studies (n=4) employed machine learning methods exclusively, focusing on classification and feature selection tasks without accompanying inferential statistics. Jeon et al. [42] developed an ensemble model using wearable IMUs and multilevel gait tasks to detect early Alzheimer’s disease. Aoki et al. [30] applied a linear Support Vector Machine (SVM) to dual-task gait data captured via Kinect to identify individuals with lower MMSE scores. Wu et al. [32] extracted cross-trial gait features from a dual-task walking and arithmetic test and used classifiers like Random Forest for cognitive prediction. Wu et al. [53] proposed a deep learning multi-modality fusion framework that integrates dual-task gait (video-derived stepping features), cognitive scores, and patch-type EEG, using a cross-attention–based fusion network and a novel loss function to automatically detect dementia.

#### 4.7.3 Combined analysis

Another subset of studies (n=9) combined statistical approaches with machine learning models, leveraging the strengths of both hypothesis-driven and data-driven frameworks. Ansai et al. [39] used both logistic regression and motion capture-derived features to identify fall predictors among MCI and AD participants, incorporating dual-task timing and TUG phase analysis. In another study, Ehsani et al. [46] extracted nonlinear features like entropy and Lyapunov exponents from upper-extremity movement and used ordinal logistic regression to classify cognitive status. Satake et al. [52] also combined a deep learning–based dual-task classification model with inferential statistical analyses to assess diagnostic accuracy and clinical associations in dementia detection.

A summary of the included studies is presented in Supplementary materials.

## 5 Discussion

In this section, we revisit our findings through the lens of the four guiding research questions, beginning with an overview of the dementia subtypes represented in the dual-task literature. Before diving into task paradigms and measurement techniques, it is crucial to understand which forms of cognitive decline have been studied and why certain subtypes dominate the evidence base.

Throughout the reviewed studies, AD overwhelmingly dominates dual-task research in dementia (17 studies), and several factors likely drive this focus. First, AD is by far the most prevalent dementia subtype, accounting for 60 to 70% of cases worldwide [57], which makes recruitment easier and findings more generalisable. Second, AD benefits from well-established clinical and biomarker-based diagnostic frameworks (amyloid Positron Emission Tomography, Cerebrospinal Fluid tau) [58]. However, other types of dementia, such as DLB, FTD or VD, often suffer from overlapping symptoms and less accessible diagnostic tests [59]. A further complication is that several studies employ the umbrella term “dementia” without specifying subtype. This choice often reflects real-world diagnostic uncertainty as many community or registry-based cohorts are identified by cognitive screening alone (e.g. MMSE or MoCA) rather than by subtype-defining neuroimaging or pathology. This also aims to capture generalisable patterns of cognitive–motor decline.

However, by not distinguishing between types of dementia, researchers may miss important differences in how each disease affects movement and cognition. For example, VD often produces early and prominent gait slowing and step asymmetry due to subcortical white-matter lesions [60], whereas FTD may spare basis gait parameters but disproportionately impair task switching and attentional allocation under dual-task conditions [36]. Likewise, mixed Alzheimer’s–vascular pathology can produce a unique blend of slowed step timing and executive dysfunction that neither pure AD nor pure VD cohorts would reveal [61]. Without subtype-level analyses, these distinct dual-task fingerprints remain hidden which can limit both our understanding and the clinical use of dual-task assessments.

Similarly, the AD-centric literature leaves important gaps. FTD and VD manifest different motor–cognitive profiles, frontally mediated behavior changes in FTD and vascular gait disturbances in VD, yet they are studied far less. Future work should explicitly recruit and study other subtypes of dementia, rigorously define cohorts by subtype whenever possible, and report subgroup-level reliability to guard against misdiagnosis. Only by broadening our lens beyond AD can we develop dual-task assessments and interventions that are sensitive to the full spectrum of eurodegenerative pathologies.

### 5.1 Types of dual-task assessments employed in dementia assessment

Our systematic review demonstrates that cognitive–motor dual-task paradigms overwhelmingly dominate the literature on dementia assessment, with 24 of 26 dual-task protocols pairing a motor task (walking or TUG) with a cognitive challenge, while only two studies examined dual motor tasks. Within the cognitive domain, 11 studies employed simple counting (forward or backward by one), seven studies used serial subtraction by three, and four studies used the more demanding serial subtraction by seven; while nine studies relied on verbal fluency and/or memory assessments. In addition, three studies incorporated more demanding arithmetic tasks, requiring real-time addition and subtraction of one- and two-digit numbers during walking, which were designed to impose sustained and adaptive cognitive load rather than fixed-sequence counting.

The predominance of arithmetic–walking dual-task conditions likely reflects both practical and pathophysiological considerations. Clinically, serial subtraction requires no special equipment, yields easily quantifiable speed and accuracy metrics, and can be administered while an individual performs normal walking, making it a natural extension of established gait assessments. More fundamentally, subtraction and other calculation tasks critically engage the dorsolateral prefrontal cortex and parietal regions so that declines in performance may serve as a sensitive marker of emerging executive-function impairment [62, 63]. In contrast, simple counting imposes a lower executive load and may miss subtle early deficits. Plummer and Eskes proposed a framework showing that simple counting imposes minimal executive load and often fails to unmask subtle dual-task deficits [64]. On the other hand, complex subtraction tasks engage attentional control, mental flexibility, and working memory which are deemed as core functions compromised in dementia [65]. Memory-based and verbal fluency dual-task paradigms, such as reciting the months of the year in reverse order, naming as many animals as possible, or sequentially reciting the alphabet, tap semantic memory and verbal fluency networks (e.g., temporal and inferior frontal cortices) [66] rather than the parietal–prefrontal axis primarily engaged by arithmetic. While these tasks place less structured demand on numerical manipulation, they can unmask deficits in lexical retrieval and category-switching that are characteristic of early frontotemporal and mixed-pathology presentations [67, 68]. However, sensitivity is strongly modulated by language proficiency and educational attainment, and these tasks typically impose weaker working-memory demands than complex subtraction [69].

Despite their ubiquity, arithmetic-based dual-task assessments introduce several biases. Firstly, they privilege numerical skill over other cognitive domains such as visualisation attention or language retrieval. Secondly, they may be unduly challenging for individuals with lower educational attainment or advanced disease. Thirdly, they tell us little about everyday multitasking scenarios, such as conversing while navigating obstacles or carrying objects while recalling directions. Memory-based and verbal fluency dual-task assessments help probe semantic fluency and retrospective-prospective recall, but these are inadequately investigated in the current literature. Dual-motor paradigms remain under-explored. Moreover, all studies restrict themselves to steady-state walking or TUG, neglecting dynamic gait challenges that include obstacle avoidance or trail-making tasks. Future work should therefore expand beyond counting backward and simple memory recall to encompass a broader spectrum of executive and attentional demands, including visuospatial matching, rapid prospective-memory cues, category fluency under load, and should integrate more ecologically valid motor tasks. For example, augmenting TUG with carrying a tray with four cups was used in a Parkinson’s disease study and showed distinct prefrontal activation patterns and gait changes between patients and healthy controls [16]. By embracing a wider variety of cognitive loads and more realistic motor scenarios, researchers can develop dual-task assessments that not only detect the earliest signs of neural dysfunction across multiple domains but also better predict everyday functional decline in people with dementia.

### 5.2 Assessment modalities and measurement devices

In the reviewed literature, five principal approaches emerged for quantifying motor function under single- and dual-task conditions in dementia assessment: IMUs, EEG sensor, force plates/pressure sensors, camera systems, and basic timing tools. Each modality balances trade-offs between precision, ecological validity, cost, and ease of use.

IMUs include accelerometers and gyroscopes affixed to the trunk, thighs, ankles, or feet and were the most popular wearable technology (nine studies). They capture raw acceleration and angular velocity waveforms at high sampling rates, from which spatio-temporal gait metrics (such as stride length, step time, cadence, variability) and turn characteristics can be derived. Compared to simple timing devices, IMUs detect subtle changes in gait rhythm and asymmetry that often precede overt slowing, and their portability allows out-of-lab, continuous monitoring [70]. In addition, IMUs benefit from their compact, body-worn form factor and wireless connectivity, allowing real-time streaming of gait data to tablets, computers, or smartphones. This noninvasive, ambulatory capability makes them especially well–suited for capturing walking performance in natural settings [71] . However, IMUs require careful sensor calibration, data post-processing pipelines to remove artifact, and some technical expertise which can cause barriers to routine clinical use [72]. As a result, ongoing efforts to develop plug-and-play calibration routines and intuitive analysis software are critical to broader clinical adoption in dementia assessment.

Instrumented walkways and standalone force platforms (used in eight studies) provide centimeter-level resolution of footfall location and millisecond-level timing, enabling extraction of center-of-pressure trajectories, weight-shift dynamics, and temporal parameters (e.g., double-support time, loading rate). These force-based features reveal balance instability during sit–stand transfers, turning phases of the TUG, and subtle postural sway that timing alone cannot capture. While exceptionally precise, force plates demand dedicated lab space and are prohibitively expensive for many clinics.

Six studies leveraged multi-camera marker-based (such as Qualisys) or markerless depth (Kinect v2) systems to reconstruct full-body kinematics. These platforms enable quantification of joint angles, trunk flexion, limb coordination, and inter-segmental timing during complex dual-task assessments. For example, a Qualisys setup can track hip, knee, and ankle excursions through a gait cycle or capture compensatory trunk sway while dual-tasking [73]. Markerless systems (Kinect) reduce setup time and cost but currently offer lower spatial resolution which can impact the accuracy of fine movement analysis [74]. This trade-off between convenience and precision is a key consideration when choosing between markerless and marker-based motion capture technologies.

One study incorporated EEG as a neurophysiological modality within a dual-task assessment framework. In this approach, patch-type EEG sensors were used to capture cortical activity during concurrent motor and cognitive tasks, enabling quantification of neural dynamics associated with cognitive load and motor–cognitive interference [53]. Unlike biomechanical sensors that infer impairment indirectly through movement variability, EEG provides direct insight into brain function, allowing extraction of spectral, temporal, and connectivity features that reflect executive processing and attentional allocation [75]. When integrated with gait and cognitive performance measures in a multimodal fusion model, EEG contributed complementary information that enhanced classification performance. However, EEG acquisition introduces practical challenges including susceptibility to motion artifacts during walking, the need for signal preprocessing pipelines, and greater setup complexity compared to purely kinematic systems [76]. Despite these constraints, EEG-based approaches highlight the potential of combining neural and motor biomarkers for more comprehensive dementia assessment.

Hand-timed stopwatches (used in five studies) remain the most ubiquitous and low-cost method for measuring total task time in the TUG and walk tests under dual-task conditions. However, they suffer from several important drawbacks: inter-rater and intra-rater variability, limited temporal resolution, and the inability to segment a trial into meaningful sub-phases (e.g., sit-to-stand, turning, straight-line gait); moreover, such assessments are labour intensive, difficult to scale, and not readily compatible with automated dementia detection workflows. As a result, stopwatch timing only provides a coarse, global motor-cost index which can be useful for rapid triage while insufficient for detailed gait or balance profiling.

Importantly, no single device captures the full spectrum of motor, postural, and neurophysiological changes associated with early cognitive decline. Despite the theoretical advantages of multimodal assessment, only one study in the reviewed literature implemented an integrated framework combining video-based gait analysis with cognitive performance measures and EEG signals [53]. This limited uptake highlights that most current approaches remain modality-specific, potentially overlooking complementary neural and motor information. Multimodal systems can leverage the complementary strengths of different sensing technologies, integrating biomechanical, temporal, and neurophysiological data, to provide a more comprehensive and robust characterisation of cognitive–motor interference than any single modality alone [77]. Other neuroimaging technologies may further strengthen multimodal frameworks. For instance, functional near-infrared spectroscopy (fNIRS) has revealed prefrontal overactivation during dual-task walking in AD, correlating with risk of falling and executive dysfunction [78]. Incorporating such neuroimaging tools alongside biomechanical measures may enhance sensitivity to dual-task costs and enable more comprehensive, ambulatory evaluations of cognitive–motor decline. Expanding future work to incorporate additional neuroimaging methods, such as EEG and fNIRS, could further enhance our ability to capture subtle cognitive and neural disruptions that precede overt motor changes in dementia.

### 5.3 Analytical methods and mechanisms

Our review reveals a clear division in how dual-task data have been analysed in dementia assessment. The majority of studies (n=13) have relied on traditional, hypothesis-driven statistical analyses, such as t-tests, ANOVAs, regression models, to quantify dual-task costs, test group differences, and correlate specific gait or cognitive features with clinical scores. These methods offer interpretability and established thresholds for significance through hypothesis testing and p-values [79] which is familiar to clinicians, and linear modelling neatly partitions sources of variance into main effects and interactions. Nonetheless, they depend on a small, investigator-selected set of predictors, assume linearity, and are underpowered to detect complex nonlinear relationships when dozens of spatiotemporal and cognitive measures are available. On the other hand, machine learning techniques have been adopted more sparingly, with only four studies in our review using machine learning exclusively, yet they promise distinct advantages for dementia detection. Machine learning models such as SVMs, random forests, and ensemble classifiers can ingest high-dimensional feature sets (e.g., multiple gait parameters and cognitive scores) and automatically identify subtle, nonlinear patterns that separate diagnostic groups and detect dementia. Compared with traditional statistical models, machine learning classifiers can flexibly weight and combine features, often yielding improved performance when relationships among variables are complex or interactive [80]. However, most machine learning applications in this field rely on relatively small datasets, single-site studies, and limited validation procedures, which increases the risk of overfitting and restricts generalisability [81]. As a result, current findings should be interpreted as exploratory rather than definitive. Furthermore, the “black-box” nature of some models can obscure which gait or cognitive features drive classification decisions, which may hinder clinical interpretability and adoption. Future studies should therefore prioritise larger multi-site datasets, rigorous external validation, and interpretable modelling approaches to strengthen the reliability and clinical relevance of machine learning–based dementia detection.

Nine studies have sought both statisitcal analyses and machine learning models by pairing data-driven feature extraction with hypothesis-driven inference. These hybrid frameworks retain statistical transparency, every selected feature can be examined for effect size and significance, while harnessing more sophisticated signal processing or machine learning-based feature selection to uncover useful predictors [82]. By arranging analysis in two steps, hybrid methods also help prevent overfitting. First, complex algorithms explore a large set of features to find patterns. Then only the strongest predictors move into a simpler, final model. As a result, the final classifier provides probabilistic estimates that can inform dementia assessment, with performance contingent on cohort, data quality, and analytic choices. The predominant use of traditional statistical methods reflects their clear interpretability and low data demands, but it risks overlooking richer, nonlinear signatures in high-dimensional dual-task recordings. By contrast, pure machine learning techniques promise greater classification accuracy and the ability to assimilate multiple gait and cognitive features simultaneously, yet they remain vulnerable to overfitting and often lack the clinical intelligibility of an ANOVA or logistic regression. Future work should therefore pursue larger cohorts to properly train and validate machine learning classifiers, couple them with feature selection methods to illuminate the most informative gait and cognitive features, and continue to refine hybrid models that blend data-driven discovery with hypothesis-driven validation.

### 5.4 Key tasks and features for dementia detection

Our pooled analysis and meta analysis findings clearly highlight certain gait parameters, particularly stride time, step length, cadence, and gait speed, serve as robust discriminators of dementia, with effects amplified under dual-task conditions. Stride time and its variability consistently emerged as sensitive indicators of cognitive–motor interference, reflecting impaired rhythmic regulation and increased attentional demands [31, 40–42, 51]. Marked reductions in step length and gait speed were observed across cognitive stages, from MCI to AD, supporting the notion of compensatory slowing and spatial restriction under heightened cognitive load [36, 37, 45]. Cadence also declined during dual-tasking, appearing particularly sensitive in differentiating moderate-to-advanced impairment rather than early-stage decline [35–37]. Although less frequently examined, swing time and its variability showed potential as markers of subtle balance disturbance and disrupted motor planning under executive demands [36, 40]. Additionally, gait analyses that integrate more sophisticated methods, such as frequency domain features, nonlinear dynamic metrics, and machine learning-driven feature extraction, could further enhance sensitivity and diagnostic accuracy, potentially revealing subtler cognitive–motor interactions and earlier indicators of dementia progression.

Upper-limb assessments, though less frequently employed, provided complementary and sensitive markers of cognitive–motor impairment in dementia. Tasks such as rapid elbow flexion consistently showed increased variability and entropy of angular velocity in individuals with MCI and early AD, reflecting subtle motor disturbances under cognitive load [46, 47]. For instance, features such as cycle-to-cycle variability, reduced range of motion, and elevated movement entropy effectively distinguished cognitively impaired groups from healthy controls, underscoring their potential value in early detection and staging of dementia severity. Similarly, balance-related features, though examined in only two studies, demonstrated notable sensitivity to cognitive impairment. Parameters such as sway path length, sway velocity, and RMS acceleration effectively differentiated AD and preclinical AD groups from cognitively healthy individuals [40, 48]. Particularly, dual-task costs in sway path length were significantly greater in preclinical AD compared to controls, highlighting increased cognitive–motor interference even in early stages.

However, the reviewed literature predominantly relies on relatively straightforward time-domain features. Notably absent are more sophisticated frequency-domain measures, such as spectral edge frequency, median power frequency, or coherence, that could uncover nuanced motor–cognitive interactions. One relevant example is the use of micro-Doppler radar and Transformer networks where Welch’s power spectral density analysis extracted frequency-based gait features that improved dementia classification accuracy compared to traditional metrics [83]. Similarly, nonlinear dynamic features such as approximate entropy and Lyapunov exponents, which quantify complexity and stability in gait patterns, have shown promise in detecting subtle gait disturbances associated with cognitive impairment [84, 85]. Additionally, few dementia studies have leveraged advanced machine learning–based feature extraction approaches, such as deep-learning embeddings or automated feature discovery directly from raw sensor signals. For instance, Zhao et al. [86] demonstrated the use of convolutional neural networks trained on raw inertial data to discover previously unrecognised gait signatures distinguishing MCI from healthy controls. Additionally, Wang et al. [87] introduced a video-based, deep learning mechanism that estimates 3D gait skeletons from monocular recordings and classifies AD vs. DLB, demonstrating that video-derived spatiotemporal gait features can effectively differentiate dementia subtypes. Such advanced analytical techniques could identify novel, highly sensitive biomarkers of dementia that extend beyond those detected by conventional statistical methods. To enhance the diagnostic accuracy and sensitivity of dual-task assessments, future research should prioritise the development and validation of these advanced analytical approaches. This includes spectral analyses, complexity-based measures, and automated deep learning pipelines trained directly on raw sensor streams. By moving beyond conventional features, these methods may uncover novel, more sensitive biomarkers capable of detecting dementia at earlier and more diverse stages of progression.

This systematic literature review has several limitations that should be considered when interpreting the findings. Although participants in the included studies had established diagnoses of dementia, the specific diagnostic procedures and the use of cognitive screening instruments varied across studies, which may introduce some variability when comparing cohorts. In addition, the meta-analysis considered dementia broadly as a single category when examining the effects of dual-task paradigms on gait outcomes. While this approach enabled synthesis across a larger body of evidence, different dementia subtypes may present distinct cognitive and motor profiles that could contribute to heterogeneity across studies. Another limitation is that different types of cognitive secondary tasks were pooled in the meta-analysis. Although cognitive tasks were pooled into broader categories (e.g., verbal fluency and arithmetic) in the meta-analysis, variations existed within each group (e.g., serial subtraction by 3s vs 7s; animal vs letter fluency). While these tasks differ slightly in cognitive demands, they all impose comparable cognitive load during walking and are commonly used in dual-task assessments. Therefore, pooling is unlikely to substantially affect interpretation, though this heterogeneity should be acknowledged as a limitation. Despite these limitations, this review provides a comprehensive synthesis of the current literature on dual-task sensor-based motion analysis for dementia detection, highlighting methodological trends, commonly used experimental paradigms, and the most informative gait features reported across studies.

Taken together, the evidence synthesised across our four research questions underscores the potential of dual-task assessments as a valuable tool for dementia detection. The literature reveals a strong reliance on cognitive–motor paradigms, particularly arithmetic-based tasks, and most studies focused on AD, likely reflecting its higher prevalence and the common practice of broadly classifying dementia as AD in clinical settings. Importantly, our meta-analysis identified several gait features, particularly stride time, step length, cadence, and gait speed under cognitive load, as the most consistently sensitive indicators of cognitive–motor decline. From a clinical perspective, these findings suggest that sensor-based dual-task gait assessments could provide an objective and non-invasive approach for supporting early detection of cognitive impairment and monitoring disease progression. Technologies such as wearable IMUs and force plates offer practical tools for capturing subtle motor deviations that may not be observable during standard clinical assessments. As such systems become more accessible, they may complement existing cognitive screening tools and contribute to more sensitive and scalable approaches for dementia screening and longitudinal monitoring. Future research should further refine task selection, explore multimodal sensing strategies, and develop robust analytical frameworks to support the translation of dual-task gait assessments into routine clinical and community-based settings across the diverse spectrum of dementia subtypes.

## 6 Conclusion

This review shows that using sensor-based dual-task assessments can reveal how cognitive tasks affect motor functions and discriminate individuals with dementia from cognitively healthy peers. Although between-study heterogeneity was substantial, reflecting differences in sensing devices, protocols, and dual-task paradigms, the direction of effects was largely consistent across studies. This suggests that cognitive–motor interference is a robust characteristic of dementia, even if the magnitude of impairment varies across settings. Across studies, dementia is consistently associated with slower gait, shorter steps, increased stride-time and step-time variability, and reduced cadence, while these are more affected under secondary cognitive loads. Arithmetic secondary cognitive tasks tend to reduce gait speed and cadence, whereas memory cognitive tasks mainly make each step take longer. Measures of turning, postural sway, and upper-limb kinematics have also been shown promising biomarkers. While inertial measurement units and pressure sensors/force platforms dominate data collection, most analyses still rely on classical statistical analyses, with growing but uneven use of machine learning pipelines. To broaden this potential, future work should include more variety of dementia subtypes, develop more complex dual-task paradigms, and include multi-modal assessments paired with validated machine learning pipelines. Embedded in routine clinic or home workflows, these assessment tools can provide scalable decision support and advance analytical quantitative measurements for dementia detection.

## Data Availability

The original contributions presented in the study are included in the article/Supplementary Material, further inquiries can be directed to the corresponding author/s.
