# Supplementary material for: A systematic review and meta-analysis on dual-task sensor-based motion analysis for dementia detection
Source: Front Digit Health. 2026 May 7;8:1728588. doi: 10.3389/fdgth.2026.1728588 (PMC13190577; doi:10.3389/fdgth.2026.1728588)
Supplement: Supplementary file 1 [file Datasheet1.pdf]

Summary of studies using dual-task in dementia detection

Table S1: Summary of studies using dual-task in dementia detection

Author	Cognitive Assessment	Participant Cohorts	Age	Single Tasks	Dual Tasks	Sensors	Analysis
Longhurst et al. 2022 Longhurst et al. (2022)	MoCA	PD: 125, AD: 127, NC: 84	74.3 ± 8.6, 75.3 ± 9.3, 70.3 ± 5.8	TUG, Arithmetic	TUG + Arithmetic	Stopwatch	Statistical
Ehsani et al. 2020 Ehsani et al. (2020)	MoCA, MMSE	NC: 35, MCI: 30, AD: 16	83.8 ± 6.9, 83.9 ± 6.9, 83.2 ± 6.6	Elbow flexion	Upper Limb + Arithmetic	IMU	Statistical, ML
Lin et al. 2016 Lin et al. (2016)	MMSE	AD: 10, NC: 10	74.0 ± 8.6, 73.8 ± 6.1	Walking	Walking + Arithmetic	Force plate, Camera	Statistical
Wu et al. 2021 Wu et al. (2021)	MMSE	HC, LC	–	Walking	Walking + Arithmetic	Force plate, Camera	ML
Ghoraani et al. 2021 Ghoraani et al. (2021)	MoCA	NC: 32, MCI: 26, AD: 20	65.13 ± 10.53, 76.81 ± 6.03, 81.40 ± 5.88	Walking	Walking + Arithmetic, Walking + Memory	Force plate	Statistical, ML
Åhman et al. 2019 Åhman et al. (2019)	MMSE	AD: 21, VD: 2, PDD: 2, Dementia: 2, FTD: 3, MCI: 52, SCI: 8	70.6 ± 7.1	TUG	TUG + Memory, TUG + Verbal Fluency	Stopwatch	Statistical
Wang et al. 2024 Wang et al. (2024)	MoCA, MMSE	NC: 38, MCI: 42, Dementia: 42	70.6 ± 7.0, 75.7 ± 7.2, 76.9 ± 7.7	Walking	Walking + Verbal Fluency	IMU	Statistical, ML
Jeon et al. 2023 Jeon et al. (2023)	MMSE	NC: 77, MCI: 68	73.5 ± 8.3, 74.8 ± 7.1	Walking	Walking + Arithmetic, Walking + Verbal Fluency	IMU	ML
Aoki et al. 2019 Aoki et al. (2019)	MMSE	HC, LC	–	Walking	Walking + Arithmetic	Force plate	ML

**Author**	**Cognitive Assessment**	**Participant Cohorts**	**Age**	**Single Tasks**	**Dual Tasks**	**Measurement Devices**	**Analysis**
Hsu et al. 2014 Hsu et al. (2014)	MMSE	AD: 21, NC: 50	61.48 ± 4.85, 59.86 ± 4.62	Walking, Standing	Walking + Arithmetic	IMU	Statistical
Venema et al. 2019 Venema et al. (2019)	MoCA	HC: 27, LC: 23	76.1 ± 5.6, 80.6 ± 6.4	TUG, Walking	TUG + Arithmetic, Walking + Arithmetic	Force plate	Statistical
Christova et al. 2022 Christova et al. (2022)	–	HC, LC	–	Walking	Walking + Arithmetic	Stopwatch	Statistical, ML
König et al. 2017 König et al. (2017)	MMSE	AD: 23, MCI: 24, NC: 22	77 ± 9, 75 ± 9, 73 ± 7	Walking	Walking + Arithmetic	IMU	Statistical
Åberg et al. 2023 Åberg et al. (2023)	MMSE	AD: 51, Dementia: 28, PDD/DLB: 11, VD: 5, FTD: 3, MCI: 38, SCI: 40, NC: 10	–	TUG	TUG + Memory, TUG + Verbal Fluency	Stopwatch	Statistical
Bovonsunthonchai et al. 2022 Bovonsunthonchai et al. (2022)	MoCA	MCI: 32, Dementia: 31, NC: 30	69.91 ± 6.96, 71.81 ± 9.46, 63.57 ± 4.78	Walking	Walking + Arithmetic	Force plate	Statistical
Tarnanas et al. 2015 Tarnanas et al. (2015)	MMSE	NC: 76, MCI: 65, AD: 86	70.06 ± 13.32, 72.63 ± 10.05, 76.59 ± 10.58	Walking	Walking + Arithmetic, Walking + Verbal Fluency	Force plate	Statistical
Ansai et al. 2019 Ansai et al. (2019)	–	MCI: 40, AD: 38	77.3 ± 6.2, 78.1 ± 6.1	TUG, Walking	TUG + Memory	Camera	Statistical, ML
Rucco et al. 2017 Rucco et al. (2017)	MMSE	NC: 20, AD: 22, FTD: 23	65.5 ± 5.4, 66.3 ± 6.0, 66.1 ± 6.2	Walking	Walking + Arithmetic, Walking + Motor	Camera	Statistical

Author	Cognitive Assessment	Participant Cohorts	Age	Single Tasks	Dual Tasks	Measurement Devices	Analysis
Bollinger et al. 2024 Bollinger et al. (2024)	MMSE	AD: 65, NC: 138	74.9 ± 5.8	Standing	Standing + Arithmetic	Force plate	Statistical, ML
Cedervall et al. 2020 Cedervall et al. (2020)	MMSE	Dementia: 25, NC: 18	77 ± 7, 72 ± 5	TUG	TUG + Verbal Fluency	Stopwatch	Statistical
Li et al. 2023 Li et al. (2023)	MoCA, MMSE	AD: 27, MCI: 35, NC: 40	67.7 ± 8.1, 65 ± 9, 60.6 ± 8.5	TUG	TUG + Arithmetic	IMU	Statistical
Ehsani et al. 2019 Ehsani et al. (2019)	MoCA	MCI: 21, NC: 79	86.4 ± 9.7, 85.1 ± 4.8	Elbow flexion, Walking	Upper Limb + Arithmetic, Walking + Arithmetic	IMU	Statistical, ML
Toosizadeh et al. 2019 Toosizadeh et al. (2019)	MoCA, MMSE	AD: 22, MCI: 34, NC: 35	84.1 ± 6.1, 83.9 ± 6.6, 83.8 ± 6.9	Elbow flexion	Upper Limb + Arithmetic	IMU	Statistical, ML
Satake et al. 2025 Satake et al. (2025)	MMSE	AD: 42, DLB: 11, MCI: 44, NC: 249	76.1 ± 10.5, 82.5 ± 6.1, 78.8 ± 6.8, 73.8±3.8	Walking	Walking + Arithmetic	Camera	Statistical, ML
Wu et al. 2025 Wu et al. (2025)	MoCA, MMSE	AD: 39, DLB: 11, MCI: 38, NC: 172	-	Walking	Walking + Arithmetic	EEG, Camera	ML
Cornish et al. 2024 Cornish et al. (2024)	MMSE	AD: 8, PD: 11, FTD: 3, CVD: 10, ALS: 3, NC: 21	72.7 ± 6.7, 65.6 ± 6.1, 63.6 ± 15.9, 68.6 ± 7.1, 56.3 ± 14, 21.9 ± 3	Walking	Walking + Arithmetic, Walking + Verbal Fluency	IMU	Statistical

REFERENCES

Longhurst JK, Rider JV, Cummings JL, John SE, Poston B, Held Bradford EC, et al. A novel way of measuring dual-task interference: the reliability and construct validity of the dual-task effect battery in neurodegenerative disease. *Neurorehabilitation and neural repair* **36** (2022) 346–359.

Ehsani H, Parvaneh S, Mohler J, Wendel C, Zamrini E, O'Connor K, et al. Can motor function uncertainty and local instability within upper-extremity dual-tasking predict amnestic mild cognitive impairment and early-stage alzheimer's disease? *Computers in biology and medicine* **120** (2020) 103705.

Lin YC, Hsu WC, Wu CK, Chang WH, Wu KPH, Wong AM. Comparison of motor performance of upper and lower extremities in dual-task tests in patients with mild alzheimer's dementia. *Aging Clinical and Experimental Research* **28** (2016) 491–496.

Wu S, Matsuura T, Okura F, Makihara Y, Zhou C, Aoki K, et al. Detecting lower mmse scores in older adults using cross-trial features from a dual-task with gait and arithmetic. *IEEE Access* **9** (2021) 150268–150282.

Ghoraani B, Boettcher LN, Hssayeni MD, Rosenfeld A, Tolea MI, Galvin JE. Detection of mild cognitive impairment and alzheimer's disease using dual-task gait assessments and machine learning. *Biomedical signal processing and control* **64** (2021) 102249.

Åhman HB, Giedraitis V, Cedervall Y, Lennhed B, Berglund L, McKee K, et al. Dual-task performance and neurodegeneration: correlations between timed up-and-go dual-task test outcomes and alzheimer's disease cerebrospinal fluid biomarkers. *Journal of Alzheimer's disease* **71** (2019) S75–S83.

Wang J, Zhou Z, Cheng S, Zhou L, Sun X, Song Z, et al. Dual-task turn velocity–a novel digital biomarker for mild cognitive impairment and dementia. *Frontiers in Aging Neuroscience* **16** (2024) 1304265.

Jeon Y, Kang J, Kim BC, Lee KH, Song JI, Gwak J. Early alzheimer's disease diagnosis using wearable sensors and multilevel gait assessment: A machine learning ensemble approach. *IEEE Sensors Journal* **23** (2023) 10041–10053.

Aoki K, Ngo TT, Mitsugami I, Okura F, Niwa M, Makihara Y, et al. Early detection of lower mmse scores in elderly based on dual-task gait. *IEEE Access* **7** (2019) 40085–40094.

Hsu YL, Chung PC, Wang WH, Pai MC, Wang CY, Lin CW, et al. Gait and balance analysis for patients with alzheimer's disease using an inertial-sensor-based wearable instrument. *IEEE journal of biomedical and health informatics* **18** (2014) 1822–1830.

Venema DM, Hansen H, High R, Goetsch T, Siu KC. Minimal detectable change in dual-task cost for older adults with and without cognitive impairment. *Journal of Geriatric Physical Therapy* **42** (2019) E32–E38.

Christova M, Strohmaier R, Fuchs-Neuhold B, Guggenberger B, Loder-Fink B, Draxler T, et al. Mixed reality prototype of multimodal screening for early detection of cognitive impairments in older adults:

Protocol development and usability study. *JMIR Research Protocols* **11** (2022) e39513.

König A, Klaming L, Pijl M, Demeurraux A, David R, Robert P. Objective measurement of gait parameters in healthy and cognitively impaired elderly using the dual-task paradigm. *Aging clinical and experimental research* **29** (2017) 1181–1189.

Åberg AC, Petersson JR, Giedraitis V, McKee KJ, Rosendahl E, Halvorsen K, et al. Prediction of conversion to dementia disorders based on timed up and go dual-task test verbal and motor outcomes: a five-year prospective memory-clinic-based study. *BMC geriatrics* **23** (2023) 535.

Bovonsunthonchai S, Vachalathiti R, Hiengkaew V, Bryant MS, Richards J, Senanarong V. Quantitative gait analysis in mild cognitive impairment, dementia, and cognitively intact individuals: A cross-sectional case–control study. *BMC geriatrics* **22** (2022) 767.

Tarnanas I, Papagiannopoulos S, Kazis D, Wiederhold M, Widerhold B, Tsolaki M. Reliability of a novel serious game using dual-task gait profiles to early characterize amci. *Frontiers in Aging Neuroscience* **7** (2015) 50.

Ansai JH, de Andrade LP, Masse FAA, Gonçalves J, de Medeiros Takahashi AC, Vale FAC, et al. Risk factors for falls in older adults with mild cognitive impairment and mild alzheimer disease. *Journal of geriatric physical therapy* **42** (2019) E116–E121.

Rucco R, Agosti V, Jacini F, Sorrentino P, Varriale P, De Stefano M, et al. Spatio-temporal and kinematic gait analysis in patients with frontotemporal dementia and alzheimer's disease through 3d motion capture. *Gait & posture* **52** (2017) 312–317.

Bollinger RM, Chen SW, Krauss MJ, Keleman AA, Kehrer-Dunlap A, Kaesler M, et al. The association between postural sway and preclinical alzheimer disease among community-dwelling older adults. *The Journals of Gerontology, Series A: Biological Sciences and Medical Sciences* **79** (2024) glae091.

Cedervall Y, Stenberg AM, Åhman HB, Giedraitis V, Tinmark F, Berglund L, et al. Timed up-and-go dual-task testing in the assessment of cognitive function: a mixed methods observational study for development of the uddgait protocol. *International journal of environmental research and public health* **17** (2020) 1715.

Li Z, Zhu J, Liu J, Shi M, Liu P, Guo J, et al. Using dual-task gait to recognize alzheimer's disease and mild cognitive impairment: a cross-sectional study. *Frontiers in Human Neuroscience* **17** (2023) 1284805.

Ehsani H, Mohler MJ, O'connor K, Zamrini E, Tirambulo C, Toosizadeh N. The association between cognition and dual-tasking among older adults: the effect of motor function type and cognition task difficulty. *Clinical interventions in aging* (2019) 659–669.

Toosizadeh N, Ehsani H, Wendel C, Zamrini E, Connor KO, Mohler J. Screening older adults for amnestic mild cognitive impairment and early-stage alzheimer's disease using upper-extremity dual-tasking. *Scientific reports* **9** (2019) 10911.

Satake Y, Taomoto D, Wu S, Godó Á, Sato S, Suzuki M, et al. The clinical significance of an ai-based assumption model for neurocognitive diseases using a novel dual-task system. *Scientific reports* **15** (2025) 13989.

Wu S, Liu J, God A, Okura F, Ikeda M, Sato S, et al. A multi-modality fusion model based on dual-task measurement for the automatic detection of early-stage cognitive impairment. *IEEE Transactions on Biomedical Engineering* (2025).

Cornish BF, Van Ooteghem K, Wong M, Weber KS, Pieruccini-Faria F, Montero-Odasso M, et al. Evaluation of a finite state machine algorithm to measure stepping with ankle accelerometry: Performance across a range of gait speeds, tasks, and individual walking ability. *Medical Engineering & Physics* **133** (2024) 104251.
